# Pancancer Fine‐Mapping of Mutational Intolerance Identifies CHEK1 as an Immunosuppressive Driver in Lung Adenocarcinoma

**DOI:** 10.1002/advs.202521265

**Published:** 2026-02-21

**Authors:** Tao Wang, Hongyu Zhao, Xiaojie Sun, Yuqi Ding, Zhipeng Zhu, Xiaotong Yu, Rongyi Zhu, Dan Wang, Kailong Li, Yang Liu, Li‐Bin Wang, Xiaolu Zhao, Baojun Suo, Hongsen Bi, Peipei Zhang, Tong Liu, Fengbiao Mao

**Affiliations:** ^1^ Institute of Medical Innovation and Research Peking University Third Hospital Beijing China; ^2^ Cancer Center Peking University Third Hospital Beijing China; ^3^ Beijing Key Laboratory for Interdisciplinary Research in Gastrointestinal Oncology (BLGO) Beijing China; ^4^ State Key Laboratory of Female Fertility Promotion Center for Reproductive Medicine Department of Obstetrics and Gynecology Peking University Third Hospital Beijing China; ^5^ National Clinical Research Center for Obstetrics and Gynecology (Peking University Third Hospital) Beijing China; ^6^ Key Laboratory of Assisted Reproduction (Peking University) Ministry of Education Beijing China; ^7^ Beijing Key Laboratory of Collaborative Innovation in Frontier Technologies for Population Quality Beijing China; ^8^ Department of Pathology Zibo Central hospital Zibo Shandong China; ^9^ Department of Biochemistry and Molecular Biology School of Basic Medical Sciences Peking University Health Science Center Beijing China; ^10^ Department of Biochemistry and Molecular Biology Beijing Key Laboratory of Protein Posttranslational Modifications and Cell Function School of Basic Medical Sciences Peking University Health Science Center Beijing China; ^11^ Department of Medical Genetics Center for Medical Genetics School of Basic Medical Sciences Peking University Health Science Center Peking University Beijing China; ^12^ Department of Cell Biology School of Basic Medical Sciences Peking University Stem Cell Research Center Peking University Health Science Center Peking University Beijing China; ^13^ Department of Gastroenterology Peking University Third Hospital Beijing China; ^14^ Department of Plastic Surgery Peking University Third Hospital Beijing China; ^15^ Key Laboratory for Neuroscience Ministry of Education/National Health and Family Planning Commission Peking University Beijing China

**Keywords:** *CHEK1*, CRISPR base‐editing screens, immunosuppression, lung adenocarcinoma, M2‐like macrophage, multi‐omics profiling, mutational intolerance

## Abstract

Mutation‐intolerant genes (MIGs), which are constrained in tumors yet variable in normal tissues, are critical for cancer survival. Herein, we developed miDriver, a computational framework using pancancer‐normal mutation contrasts to identify 1,020 MIGs across 8,096 tumors of 13 cancer types. Strikingly, MIGs are highly associated with synthetic lethality, cell‐cycle progression, and clinical outcome. CRISPR screening reveals MIGs, especially *CHEK1*, as cancer‐specific vulnerabilities, whose suppression impairs tumor proliferation and migration. Single‐cell transcriptomics reveals a CHEK1‐high subpopulation exhibiting stem‐like and immune‐suppressed features, linking tumor‐intrinsic fitness to microenvironment remodeling. Multiplexed immunofluorescence revealed that CHEK1 and MIF are co‐expressed in tumor cells, and CHEK1‐high tumor cells exhibit closer spatial proximity to M2‐like macrophages. Mechanistically, CHEK1 promotes p53 phosphorylation to upregulate MIF expression and secretion, thereby driving M2‐like macrophage polarization via the MIF–CD74 axis. In vivo, targeting the CHEK1–MIF axis (particularly CHEK1) broadly reverses immunosuppression. Clinically, higher tumor CHEK1 levels are associated with poorer response to anti‐PD‐1 therapy. Exemplified by *CHEK1*, these findings establish MIGs as dual therapeutic targets capable of simultaneously disrupting tumor‐intrinsic fitness and remodeling the immunosuppressive niche. This work proposes a novel paradigm for selectively targeting MIGs to eliminate aggressive tumor subclones while minimizing toxicity to normal cells.

## Graphical abstract

This study identifies mutation‐intolerant genes (MIGs), which are mutationally constrained in tumors despite normal‐tissue variability. Using miDriver, the authors pinpoint MIGs essential for tumor‐intrinsic fitness and immune evasion. Focusing on CHEK1, they show it drives tumor fitness and sculpts an immunosuppressive niche via the MIF–CD74 axis. Targeting MIGs like CHEK1 presents a dual‐therapy strategy to eliminate tumors.

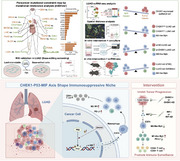

## 1 Introduction

While mutational constraint has been widely used to pinpoint disease‐associated fitness genes, its application in cancer genomics is far more difficult, owing to the complex selective pressures in tumors. CRISPR‐Cas9‐based saturation mutagenesis studies of key cancer genes, such as *BAP1* [1], *RAD51C* [2], *TP53* [3], and *BRCA2* [4, 5], have demonstrated that the majority of functional mutations are selectively intolerant to tumor cells, underscoring their therapeutic relevance. However, genome‐wide systematic identification of all genes exhibiting such mutation intolerance remains technically and analytically challenging. Interestingly, recent large‐scale sequencing of normal human tissues has revealed a substantial enrichment of somatic mutations in certain genes, suggesting unexpected tolerance to genetic disruption in non‐malignant contexts [6, 7, 8, 9]. Despite being highly mutated in normal tissues, certain genes show remarkable intolerance to missense mutations in cancers. This pattern implies negative selection and tumor‐specific essentiality, defining them as mutation‐intolerant genes (MIGs). This distinct mutational signature, emerging under oncogenic selection, implies the existence of synthetic lethal interactions and reveals previously unrecognized therapeutic vulnerabilities [10, 11, 12].

Synthetic lethality has been widely used in cancer therapy, enabling the targeted elimination of malignant cells by exploiting their specific genetic vulnerabilities [12]. This approach gained clinical validation through the success of PARP inhibitors in BRCA1/2‐mutant breast cancer [13]. Multiple candidate targets are currently advancing through therapeutic development, including *WRN* [14], USP1 [15], *PKMYT1* [16, 17], *POLQ* [18], and *PRMT5* [19], while *TPX2* [20], *WEE1* [21], and *CDK12* [22]. Recent discoveries of novel synthetic lethal pairs (e.g., CDS2‐CDS1 in uveal melanoma [23] and RBM10‐WEE1 in LUAD [24]) and complex‐dependent vulnerabilities (e.g., PELO‐HBS1L in SKI‐dysfunctional tumors [25]) continue to expand this landscape. However, the contribution of scarcely mutated MIGs to synthetic lethal networks is poorly understood. These genes constitute a class of neglected therapeutic targets, thus holding high potential to broaden the scope of precision oncology.

To systematically evaluate the functional significance of MIGs across human cancers, we developed miDriver, a computational framework designed to identify MIGs by integrating somatic mutation patterns from large‐scale cancer genomes (TCGA [26]) and normal tissue cohorts (SomaMutDB [27]). Pancancer mutational intolerance analysis revealed MIGs to be significantly enriched in synthetic‐lethal interactions, core oncogenic pathways, and prognostic biomarkers. Consistently, single‐cell transcriptomic profiling across cancers demonstrated that these genes are active in tumor cells, driving both proliferation and immunosuppression. We employed a CRISPR base‐editing screen to functionally validate a curated set of mutations in MIGs from LUAD‐matched normal lung tissue, as well as top candidates from histologically matched normal tissues of other major cancer types. The screen confirmed that disruption in the majority of these mutations in MIGs induces synthetic lethality selectively in cancer cells while sparing non‐malignant cells. Single‐cell Perturb‐seq analysis demonstrated significant downregulation of the targeted MIGs and concomitant suppression of oncogenic pathways. Analysis of LUAD single‐cell RNA‐seq data identified a distinct CHEK1‐high tumor subpopulation that drives macrophage M2 polarization. Mechanistically, CHEK1 phosphorylates p53 to upregulate MIF expression and secretion, thereby establishing an immunosuppressive niche. Targeting this CHEK1–MIF axis, particularly CHEK1, broadly reversed immunosuppression, confirming its central role. Collectively, these findings nominate MIGs as a class of high‐value therapeutic targets, offering a promising strategy for cancer‐specific treatment with minimal toxicity to normal tissues due to their inherent mutation tolerance in healthy cells.

## 2 Results

### 2.1 Detection and Clinical Significance of Mutation‐Intolerant Genes

**FIGURE 1:**
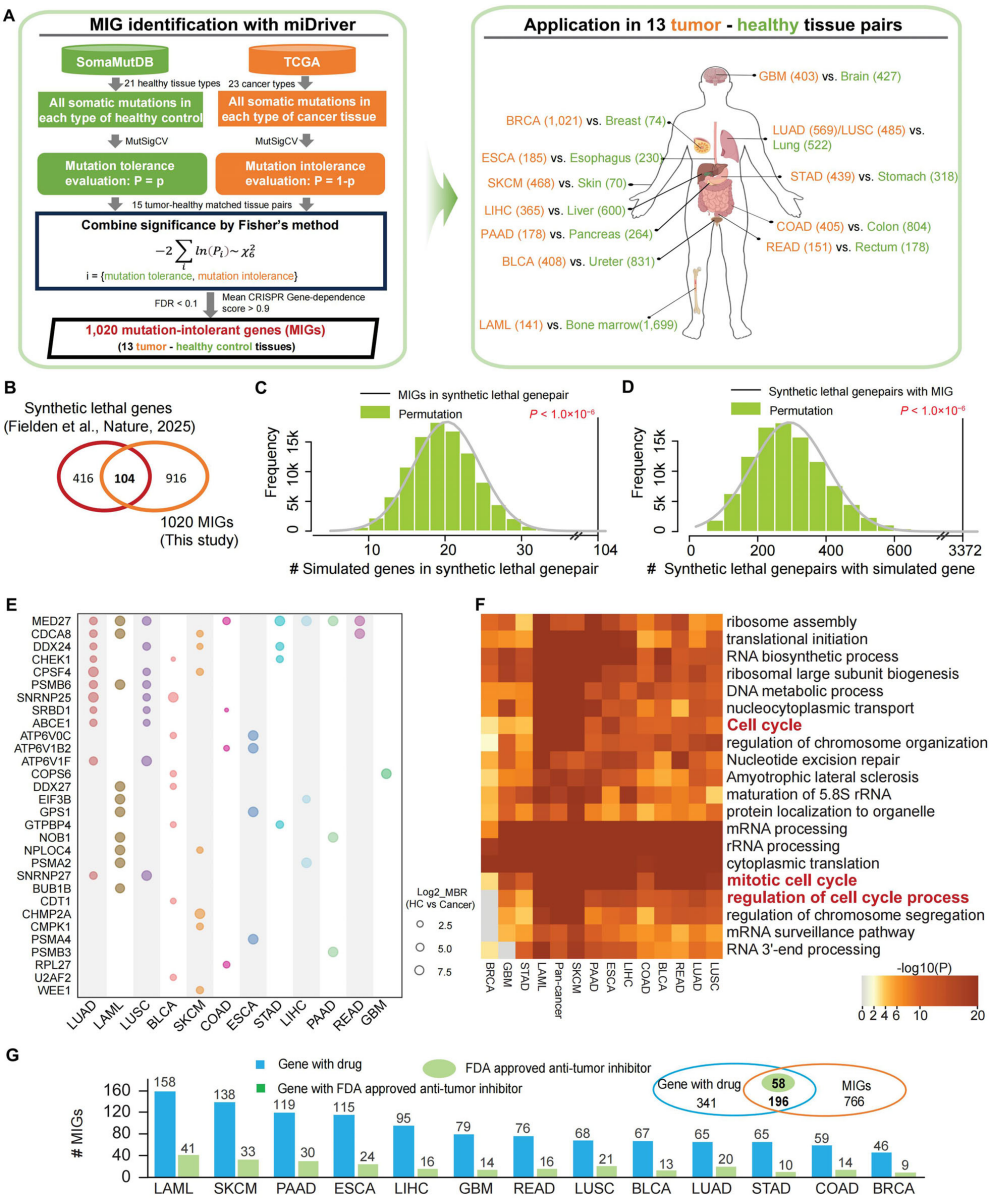
Identification of MIGs in cancers and their clinical significance. (A) Workflow for identifying MIG in tumors. First, somatic mutations from 21 healthy tissues and 23 cancer types were analyzed to evaluate gene mutation tolerance using MutSigCV. Subsequently, Fisher's method was applied to access the overall significance. Finally, gene with a mean CRISPR gene‐dependence score > 0.9 and FDR < 0.1 in corresponding cancer cell lines was classified as MIGs, resulting in 1020 MIGs in 13 tumor‐healthy tissue pairs (as detailed in Methods). MIG, mutation‐intolerant gene; TME, tumor microenvironment; (B) Overlap between MIGs identified in this study and previously reported synthetic lethal (SL) gene pairs (Fielden et al., *Nature*, 2025); (C) MIGs show significant enrichment among known SL genes (*p* < 1.0 × 10^−6^) and form more SL pairs than random expectation (D) (*p* < 1.0 × 10^−6^). The *p*‐value was calculated using a permutation test with 1 000 000 iterations; (E) Distribution of the top 30 MIGs across different cancer types, which remained mutation‐free within tumors but exhibited mutational tolerance in respective healthy controls (as detailed in Methods). MBR, ratio of mutational burden between healthy control (HC) and cancer patients; (F) Heatmap of enrichment results for MIGs revealed significant enrichment in cell cycle‐related functions across different cancer types. MIG, mutation‐intolerant gene.

Consistent with previous studies [6, 28], we detected significantly mutated genes (SMGs) not only in tumor tissues but also in morphologically normal tissues from tumor‐free organs (Figure S1A). Among 335 SMGs identified in 6603 healthy control tissues across 21 organs, 22 overlapped with SMGs recurrently mutated across multiple cancer types (Figure S1A–C; Table S1). Notably, the vast majority (>93%) of SMGs found in healthy controls were distinct from those observed in tumors, indicating distinct mutation signatures between healthy and cancerous tissues (Figure S1D,E). Leveraging these observations, we developed miDriver, a systematic computational framework for identifying Mutation‐Intolerant‐Genes (MIGs) in tumors based on somatic mutation patterns derived from tumor and matched normal tissues. Using this approach, we identified 1,020 MIGs across 13 cancer types (Figure 1A; Table S1). Notably, these MIGs were significantly enriched in experimentally validated synthetic lethal (SL) gene pairs (*p* < 1.0 × 10^−6^, permutation test, Figure 1B,C; Table S1). Comparative analysis further revealed that MIGs were significantly enriched for synthetic lethal interactions compared to random expectation (*p* < 1.0 × 10^−6^, permutation test, Figure 1D). Among the top 30 MIGs are key drivers, including *MED27*, *CDCA8*, *DDX24*, *CHEK1*, and *CPSF4* (Table S1), which have been directly implicated in tumor progression across various cancers [29, 30, 31, 32, 33, 34].

To elucidate the biological functions of the identified MIGs, we performed pathway enrichment analysis and revealed their significant enrichment in cell cycle‐related processes, accounting for ∼30% of all MIGs (Figure 1F; Figure S1F; Table S1). These MIGs were also enriched for known drug targets (*p* = 2.70 × 10^−163^, hypergeometric test) and associated with FDA‐approved anti‐tumor inhibitors (*p* = 3.85 ×  10^−5^, hypergeometric test, Figure 1G; Table S1). We next evaluated the correlation between the expression of MIGs in tumors and patient's overall survival. Across cancer types, approximately 12% (40/334) of MIGs were significantly associated with patient survival, among which 40% (16/40) were classified as cell cycle genes (Figure S1G; Table S1). Skin cutaneous melanoma (SKCM) contained the highest number of survival‐related MIGs, followed by pancreatic Adenocarcinoma (PAAD) and lung adenocarcinoma (LUAD). Overall, these results demonstrated that MIGs were significantly enriched in well‐known cancer pathways and were closely associated with clinical outcomes, underscoring their broad clinical relevance.

### 2.2 Prognostic Implications of MIGs in Multiple Cancer Types

**FIGURE 2:**
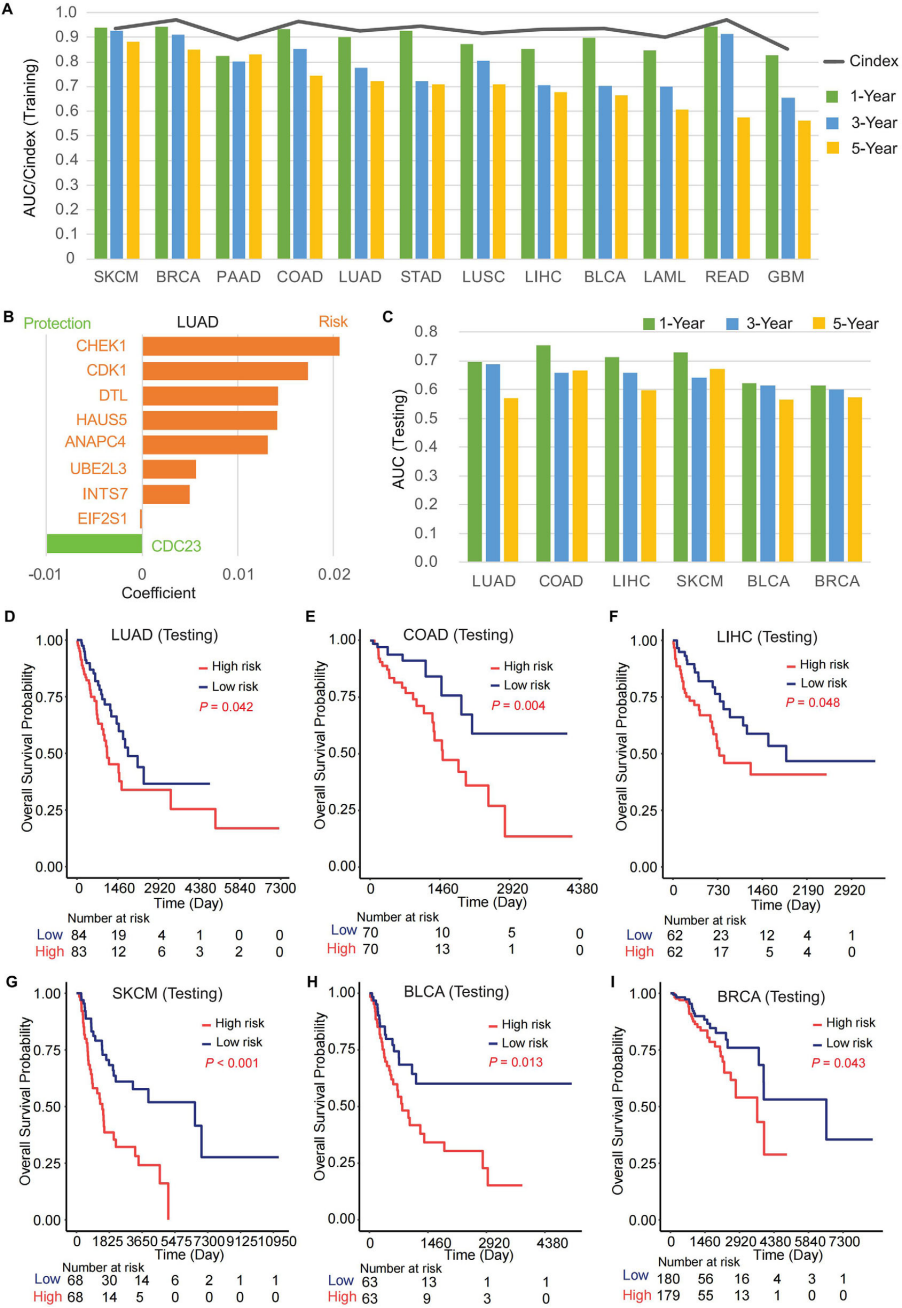
Systematic evaluation of the prognostic implications of MIGs in various types of cancer. (A) Diagnostic accuracy of the constructed prognostic model based on cell cycle‐related MIGs was assessed using time‐dependent AUC and C‐index in the training set across various types of cancer. (B) Coefficients of the selected MIGs by the constructed LASSO model in LUAD tumors indicated that most of the selected MIGs were risk factors associated with cancer prognosis. (C) Evaluation of the prognostic potential of MIGs in testing cohorts selected for their relatively large sample sizes across six cancer types. (D‐I) Kaplan–Meier overall survival curves between patients with a lower CMPS (low risk) and those with a higher CMPS (high risk). MIG, mutation‐intolerant gene. AUC, area under the receiver operating characteristic curve. CMPS, CellCycle MIG‐related prognostic risk score. The median value of CMPS was used for grouping patients into CMPS high or low‐risk groups in each cancer type. The log‐rank test was used for survival analysis.

Given the pronounced enrichment of MIGs in cell cycle functions, we hypothesized this biologically coherent subset holds clinical relevance and therefore applied LASSO Cox regression to assess its prognostic value across multiple cancer types [35]. Models were constructed using 10‐fold cross‐validation optimized for the minimum λ to mitigate overfitting. The resulting MIG‐based signatures significantly predicted overall survival (OS) in diverse cancers, achieving an average C‐index of 0.92. Time‐dependent ROC analysis yielded average AUC values of approximately 0.89, 0.79, and 0.72 for 1‐, 3‐, and 5‐year survival, respectively (Figure 2A). Each cancer‐specific model selected an average of 14 prognostic MIGs. Notably, many high‐risk MIGs identified in these models, including *DTL* [36, 37], *USP39* [38, 39, 40], *CHMP2A* [41], *FBX05* [42, 43], *TINF2* [44, 45], and *CHEK1* [46], have previously been implicated in tumor progression and prognosis (Figure S2A). Among these, *DTL* [36], *USP39* [38], and *CHMP2A* [41] are known to modulate the tumor microenvironment to support tumor progression.

The majority of the prognostic MIGs were associated with poorer OS across cancer types (Table S2). For example, approximately ∼89% (8/9) of the prognostic MIGs selected by the final LASSO model were risk factors for OS in LUAD (Figure 2B). Using the resulting linear predictor, we computed a CellCycle MIG‐related Prognostic Score (CMPS) for each patient across all cancer types. As expected, high‐risk patients defined by CMPS exhibited significantly worse survival outcomes than low‐risk patients across multiple malignancies (*p* < 0.0001, Figure S2B). To validate the findings, we constructed models using 70% of the data for each cancer type, reserving 30% as an independent test set. The MIG‐based signature robustly predicted overall survival in all validation cohorts, with results for the six largest cohorts (LUAD, COAD, LIHC, SKCM, BLCA, BRCA) presented in Figure 2C and for all others in Figure S2C. Consistently, higher CMPS was associated with poorer survival in all tested cancer types (*p* < 0.0001, Figure 2D–I). Overall, these results underscore the broad and robust prognostic implications of MIGs across human cancers.

### 2.3 MIGs Drive Tumor Progression by Promoting an Immunosuppressive Microenvironment

Since several MIGs have previously been implicated in tumor progression through modulation of the TME [36, 38, 41], we assessed whether the CMPS were associated with altered immune cell infiltration. Tumors with low‐CMPS exhibited significant enrichment of anti‐tumor immune cells, including M1 macrophages, CD8 T cells, and memory B cells, across multiple cancer types (Figure S3A; Table S3). Consistent with this pro‐immunogenic profile, tumors with high‐CMPS were associated with a pro‐tumor immune landscape, marked by expanded M2 macrophage infiltration and reduced abundance of M1 macrophages (Figure S3B).

**FIGURE 3:**
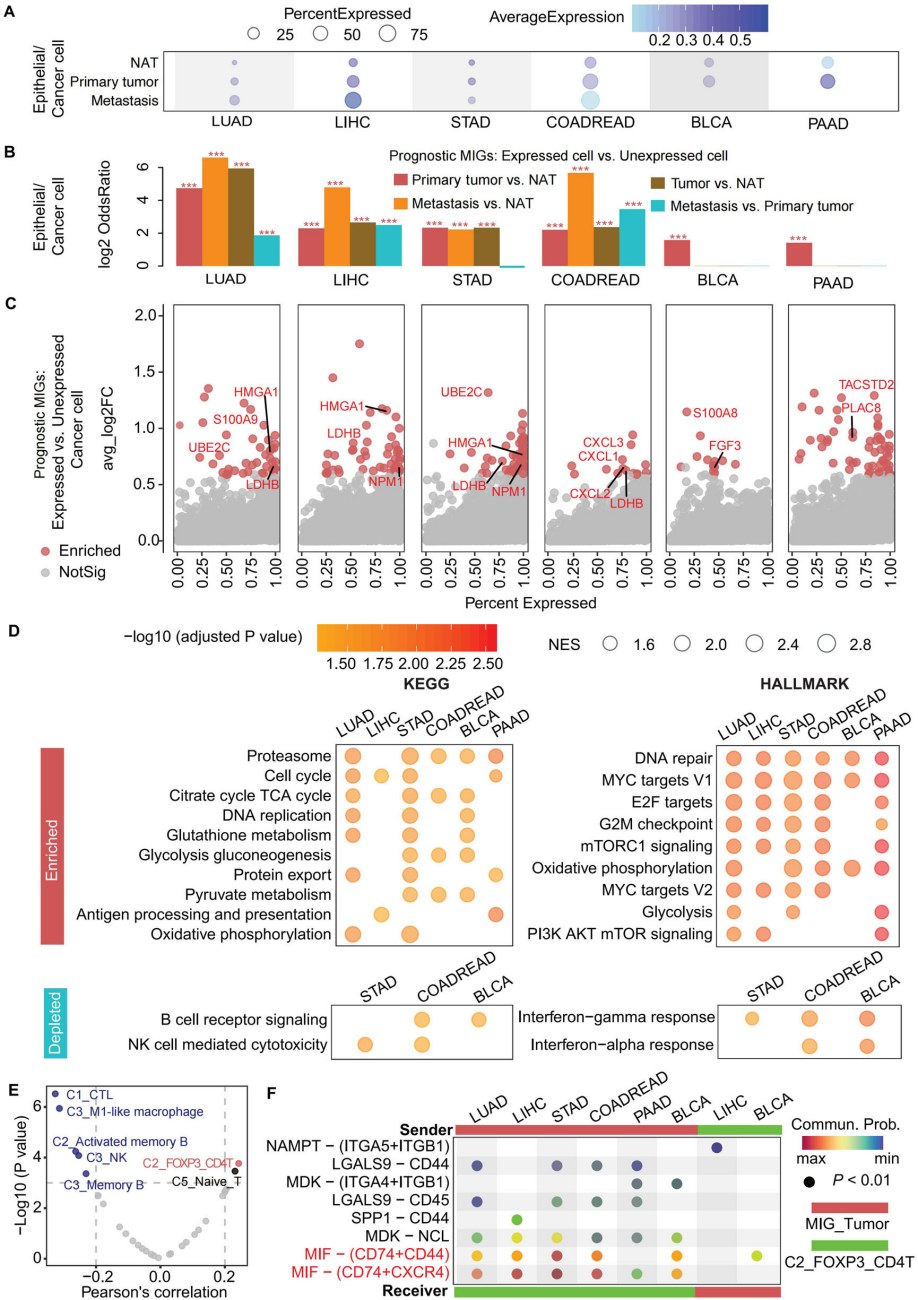
MIGs inducing an immunosuppressive microenvironment via MIF signaling to promote tumor progression. (A) The expression pattern of prognostic MIG in cancer cells (both primary tumors and metastatic tumors) and epithelial cells from NATs across various types of cancers. The size of the dot represents the percentage of expressed cancer or epithelial cells. The color of the dot represents the scaled average expression level of prognostic MIGs. (B) Comparison of the percentage of prognostic MIG‐expressing cancer cells across each kind of primary tumors, metastatic tumors, and NATs using a Chi‐square test. ^***^, *p* < 0.001; The *Y*‐axis represents the log2‐transformed odds ratio. (C) Differential expression results between prognostic MIG‐expressing cancer cells and the remaining cancer cells. The *Y*‐axis represented the average log2‐transformed fold change (FC) of gene expression between two groups, while the *X*‐axis represented the expression percentage of each gene in prognostic MIG‐expressing cancer cells. (D) GSEA enrichment analysis of the significantly dysregulated KEGG pathways and HALLMARKs in prognostic MIG‐expressing cancer cells across various cancer types. The size of the dot represented the normalized enrichment score (NES). The color of the dot represented the adjusted *p*‐values. (E) The co‐expression patterns of prognostic MIG‐expressing cancer cells with other immune cell populations based on Pearson’ correlation analysis. |R| > 0.2 and *p* < 0.001 were considered as the significant threshold. (F) Cell–cell communication between prognostic MIG‐expressing cancer cells and their significantly co‐enriched C2_FOXP3_CD4T cells. The top five significantly enriched intercellular interactions for each cancer type were displayed. CTL, cytotoxic CD8 T cells; NK, Natural killer cells. The color of the dot represents the communication probability.

To gain mechanistic insight into how MIGs shape the TME and support tumorigenesis, we integrated 15 publicly available scRNA‐seq datasets spanning six cancer types, comprising 280 samples and 874 132 cells (Table S4). After rigorous quality control, batch correction, and cell‐type annotation, we evaluated the expression of prognostic MIGs across epithelial cells from normal adjacent tissues (NATs), primary tumors, and metastatic lesions (Figure S4). Prognostic MIGs were consistently upregulated in tumor cells compared to NATs across all cancer types (Figure 3A,B). Notably, in LUAD, LIHC, STAD, and COADREAD, these genes were further elevated in metastatic tumors relative to primary tumors (Figure 3B).

Among the prognostic MIG‐expressing cancer cells, we observed significant upregulation of established oncogenes including *LDHB* [47], *HMGA1* [48], *UBE2C* [49, 50, 51], *NPM1* [52], *CXCL1/2/3* [53, 54], *S100A9* [55, 56], *S100A8* [56], *FGF3* [57], *PLAC8* [58], and the drug target *TACSTD2* [59, 60] (Figure 3C). Pathway enrichment analysis revealed activation of pro‐tumorigenic processes such as cell cycle, MYC targets, E2F targets, mTORC1 signaling, PI3K/AKT/mTOR signaling, and glycolysis. Conversely, immune‐effector pathways critical for tumor killing, such as B cell receptor signaling, NK cell‐mediated cytotoxicity, and interferon‐alpha/gamma responses, were suppressed (Figure 3D).

We next correlated the abundance of prognostic MIG‐expressing cancer cells with immune cell proportions via Pearson correlation analysis. High expression of prognostic MIGs was associated with reduced infiltration of anti‐tumor immune populations, such as cytotoxic CD8 T cells (CTLs), M1‐like macrophages, natural killer cells, and memory B cells. In contrast, *FOXP3* ^+^ regulatory T cells (C2_FOXP3_CD4T), which exert immunosuppressive functions [61], were significantly enriched (Figure 3E). This T cell population also exhibited elevated expression of the co‐inhibitory receptors *TIGIT* and *CTLA4* [62], which dampen T and NK cell activity (Figure S4J). Cell–cell communication analysis further indicated that prognostic MIG‐expressing cancer cells secrete macrophage migration inhibitory factor (MIF), which engages CD74/CD44 receptors on immune cells to promote immunosuppression (Figure 3F). This MIF‐mediated pathway has been previously linked to immune evasion in breast and pancreatic cancer [63, 64]. Taken together, these results suggest that prognostic MIGs promote an immunosuppressive microenvironment, thereby enabling immune evasion and supporting tumor progression.

### 2.4 MIGs Drive Tumor Progression in LUAD Cancers by Remodeling the TME

**FIGURE 4:**
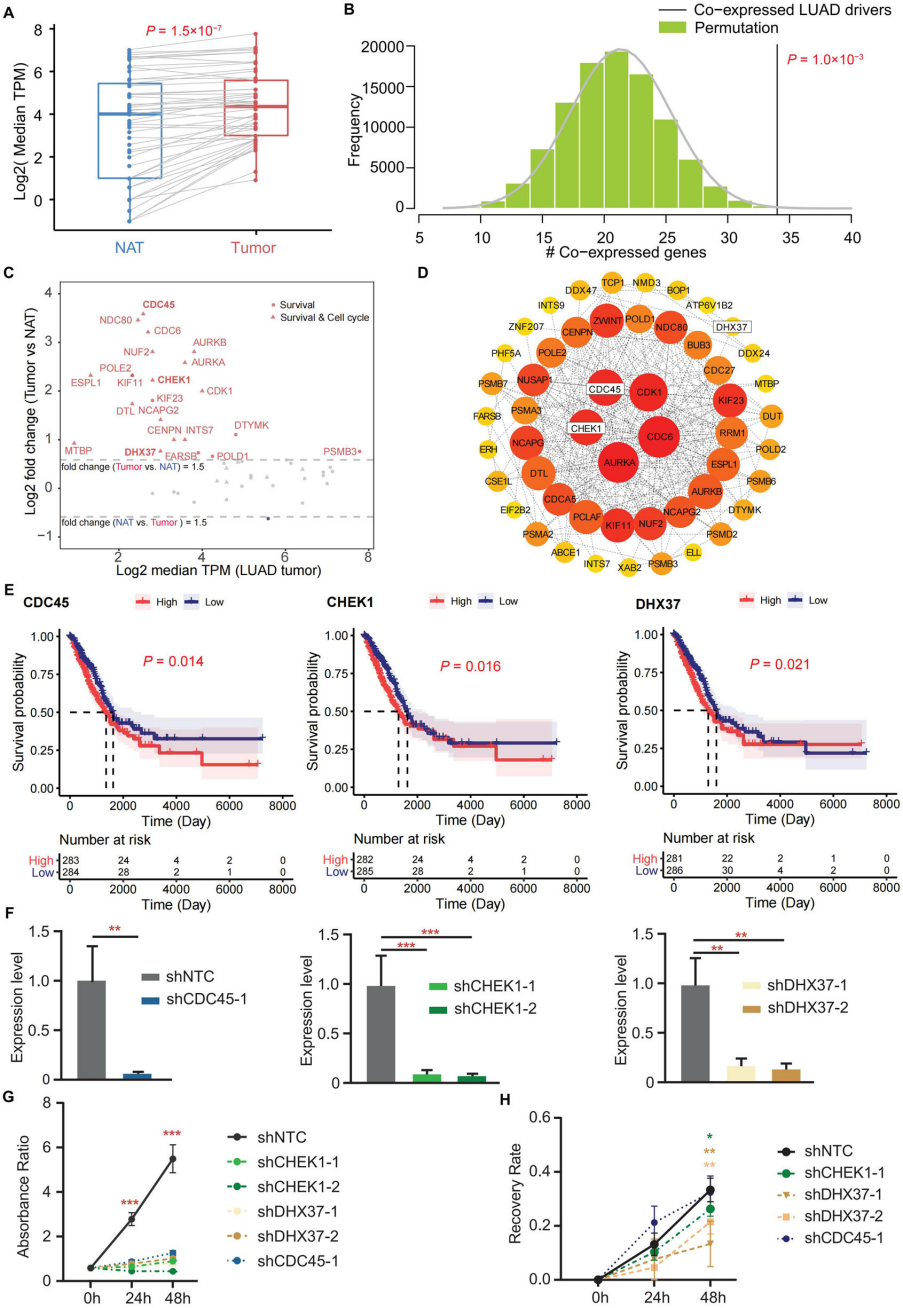
Significant clinical importance of MIGs identified in LUAD. (A) The expression levels of MIGs were significantly higher in tumor tissues compared to NAT in LUAD. The Wilcoxon‐rank sum test was used for *p*‐value calculation. (B) MIGs were significantly co‐expressed with known LUAD driver genes (permutation test, *p* < 0.001, 1 000 000 iterations). Gene pairs with |R| > 0.5 and *p* < 1.0 × 10^−8^ were considered as co‐expressed genes. (C) The differential expression of each survival‐related MIGs between tumor tissues and NAT. (D) The PPI network of survival‐related MIGs was constructed using the STRING database and analyzed in Cytoscape (version 3.10.1); Both node size and color intensity scale with degree centrality, where larger diameters and deeper red hues indicate higher connectivity within the network. (E) Survival plots showed that patients with a high expression level of *CDC45*, *CHEK1*, and *DHX37* genes exhibited significantly worse overall survival compared to those with a correspondingly low expression level. The Log‐rank test was used for *p*‐value calculation. (F) The expression of *CDC45*, *CHEK1*, and *DHX37* genes was significantly downregulated in the respective gene knockdown A549 cells with shRNA targeting, as quantified by qPCR. (G) CCK8 assays demonstrated that the downregulation of *CDC45*, *CHEK1*, and *DHX37* genes significantly reduced cell proliferation in the A549 cell line. (H) Wound healing assays revealed that the downregulation of *CHEK1* and *DHX37* genes significantly reduced the migratory ability of A549 cells. The Dunnett's multiple comparisons test was used for *p*‐value calculation. MIG, mutation‐intolerant gene; NAT, normal adjacent tissue; LUAD, Lung Adenocarcinoma; shNTC, non‐targeting control shRNA; ^*^, *p* < 0.05; ^**^, *p* < 0.01; ^***^, *p* < 0.001.

Among the top three cancers enriched in MIGs, LUAD had matched gene expression profiles available from both tumor and NAT, making it suitable for a detailed investigation into the functional relevance of MIGs. In LUAD tumors, MIGs were significantly upregulated compared to NAT (*p* = 1.50 × 10^−7^, paired Wilcoxon rank‐sum test, Figure 4A; Table S5). Furthermore, MIGs showed significant co‐expression with known LUAD driver genes (*p* = 1.03 × 10^−3^, permutation test, Figure 4B; Table S5), implicating their potential involvement in tumor progression. Integrated analysis of tumor transcriptomes and protein‐protein interaction networks pinpointed *CDC45*, *CHEK1*, and *DHX37* as candidate genes for further functional validation. All three genes were significantly overexpressed in tumor tissues compared to NATs (Figure 4C; Figure S5A), with *CHEK1 and CDC45* identified as hub regulators within the interaction network (Figure 4D; Table S5). Moreover, higher expression of these three genes was associated with significantly worse OS (*p* < 0.05, Figure 4E). Further, each candidate also exhibited significant co‐expression with *MKI67*, a marker of cell proliferation (Figure S5B–D).

To functionally validate the oncogenic role of MIGs in LUAD, we generated A549 cell lines with stable knockdown of *CDC45*, *CHEK1*, or *DHX37* using shRNA plasmid transfection. Endogenous expression of each gene was significantly reduced compared to non‐targeting control shRNA (shNTC)‐transfected cells (Figure 4F; Table S5). We then evaluated the impact of gene suppression on cellular proliferation and migration using CCK‐8 and wound healing assays, respectively. Strikingly, knocking down any of the three genes markedly inhibited LUAD cell proliferation (*p* < 0.0001, Dunnett's multiple comparisons test; Figure 4G) and reduced cell migration (*p* < 0.05, Dunnett's multiple comparisons test; Figure 4H; Figure S5E; Table S5). Our results suggested a positive association between these MIGs and tumor progression.

To further elucidate how MIGs drive tumor progression, we performed RNA‐seq analysis on LUAD cells following gene knockdown. Each sample yielded an average of 6.55 GB of raw data with a unique mapping rate of 90.09% (Table S6). Principal component analysis (PCA) confirmed high sample quality and clear group separation (Figure S6A–C). Differential expression analysis (DESeq2) revealed 1673, 2115, and 1990 significantly upregulated protein‐coding genes, and 1643, 1535, and 2517 significantly downregulated protein‐coding genes for the shCHEK1, shDHX37, and shCDC45 knockdowns compared to shNTC, respectively (Table S6). Among these, 279 upregulated and 183 downregulated genes were shared in all three knockdowns, including established oncogenes and tumor suppressors (Figure S6D). *CHEK1* knockdown suppressed the expression of *TSPAN8* and *SOX2*, both associated with the promotion of cancer stemness (Figure S7A). *TSPAN8* has been linked to tumor progression and metastasis in LUAD [65], while *SOX2* is a key regulator of stem cell pluripotency and tumorigenesis [66]. Additionally, wild‐type cells showed elevated expression of the tumor‐promoting genes *BPIFB1* [67] and *MUC13* [68], and reduced the expression of the tumor suppressors of *GPR27* [68] and *FOXL2* [69], relative to *CHEK1*‐knockdown cells (Figure S7A).

Gene Set Enrichment Analysis (GSEA) further indicated that differentially expressed genes (DEGs) following *CHEK1* knockdown were enriched in pathways related to cell cycle, telomere function, E2F, and MYC targets, which are critical to cancer stemness and tumor microenvironment (TME) modulation [70] (Figure S7B; Table S6). Similar dysregulation was observed after *DHX37* knockdown, including suppression of NOTCH signaling, which is involved in stemness maintenance [71] and TME remodeling [72] (Figure S7C,D). Likewise, *CDC45* knockdown led to broad transcriptomic changes consistent with those in *CHEK1* and *DHX37* knockdowns, with notable suppression of glycolysis‐related pathways, known to support tumor progression and immune evasion [73] (Figure S7E,F). Together, these results indicate that *CHEK1*, *DHX37*, and *CDC45* collectively facilitate LUAD progression by modulating cancer stemness and the TME (Figure S7G).

### 2.5 CRISPR Screening Unveils MIG Disruption as a Synthetic‐Lethal Vulnerability in LUAD

**FIGURE 5:**
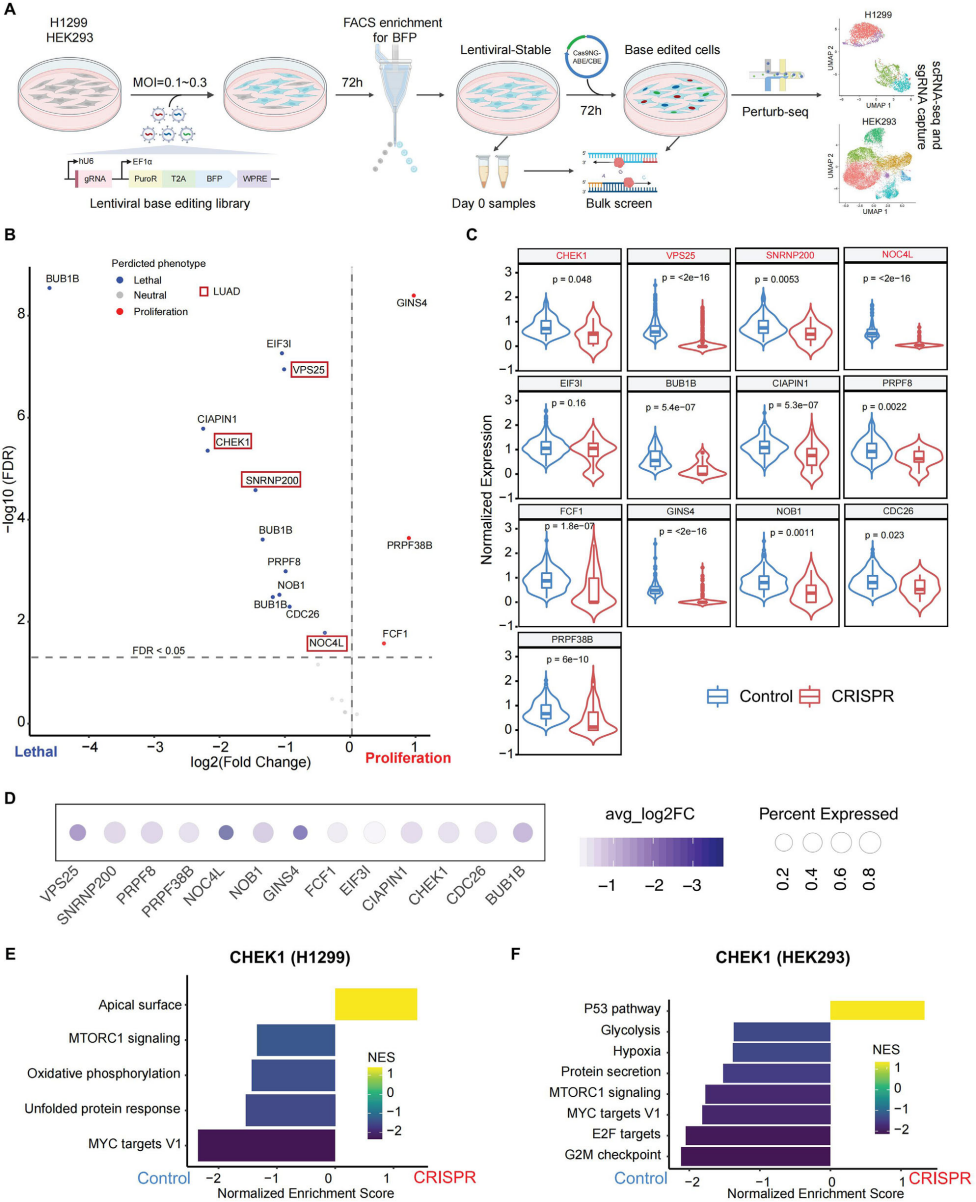
Integrated functional genomics identifies MIG disruption as a therapeutic vulnerability in LUAD. (A) Schematic of the screening workflow: the integration of bulk CRISPR screening with Perturb‐seq identifies the impact of genetic perturbations on cell phenotype and the underlying transcriptomic changes. (B) Analysis of MIG editing effects from the CRISPR screen, normalized to day 0 sgRNA abundance (generalized linear model; FDR < 0.05). (C) Box plot of targeted gene expression levels in edited cells and non‐target control cells. Statistical significance was determined by the Wilcoxon rank‐sum test. (D) Dot plot visualization of targeted gene expression in edited cells. Dot size indicates the percentage of cells expressing the gene; color scale represents the mean log2 fold change relative to control cells. (E‐F) GSEA shows downregulation of oncogenic pathways following CHEK1 targeting in H1299 (E) and HEK293 cells (F), as shown by GSEA.

However, the systematic functional characterization and therapeutic potential of MIGs in LUAD remain largely unexplored. To bridge this gap, we designed a CRISPR‐based editing screen targeting MIG variants prevalent in healthy human tissues. Parallel screening in LUAD (H1299) and non‐malignant (HEK293) cell lines enabled the identification of mutations that are selectively lethal in cancer cells while tolerated in healthy controls (Figure 5A; Table S7).

To decipher the transcriptional alterations following MIG perturbation and to simultaneously assess their impact on cell fitness, we performed Perturb‐seq in LUAD cells at endpoint, and successfully profiled 10 000 high‐quality single cells, with a median of 3,500 genes detected per cell (Figure S8A–D). We then modeled the relationship between the initial sgRNA abundance (Day 0) and the resulting cell population changes in the Perturb‐seq data using a generalized linear model (GLM). Our integrated model identified a synthetic‐lethal vulnerability for the majority of MIG perturbations in LUAD cells (Figure 5B; Table S7). Notably, this vulnerability was not limited to LUAD‐derived MIGs such as *VPS25* and *CHEK1*, as a substantial fraction of MIGs identified in other cancer types also exhibited synthetic lethality in LUAD. Furthermore, MIG‐edited cells universally showed significantly reduced expression of their corresponding genes (Figure 5C,D), providing direct transcriptional proof of on‐target editing and functional validation of the hits. This pattern reveals shared genetic dependencies across cancer types and underscores the therapeutic potential of targeting these core vulnerabilities.

GSEA analysis revealed a significant suppression of MYC targets and mTORC1 signaling in CHEK1‐edited LUAD cells, pathways critical for their survival (Figure 5E). A similar downregulation of these oncogenic pathways was observed following *VPS25* and *NOC4L* editing (Figure S8E,F). Notably, *CHEK1* editing in non‐malignant (HEK293) cells resulted in a broader attenuation of proliferation‐associated programs, including MYC and E2F targets, G2M checkpoint, and mTORC1 signaling (Figure 5F). Critically, this convergent transcriptional perturbation proved lethal to LUAD cells but was tolerated by non‐malignant cells, revealing a cancer‐specific vulnerability. Collectively, our data establish MIG perturbation as a synthetic‐lethal target in LUAD. The cancer‐specific lethality highlights its therapeutic promise and suggests a favorable safety profile.

### 2.6 CHEK1 Drives LUAD Progression by Activating Both Tumor‐Intrinsic Fitness and Immune Evasion

**FIGURE 6:**
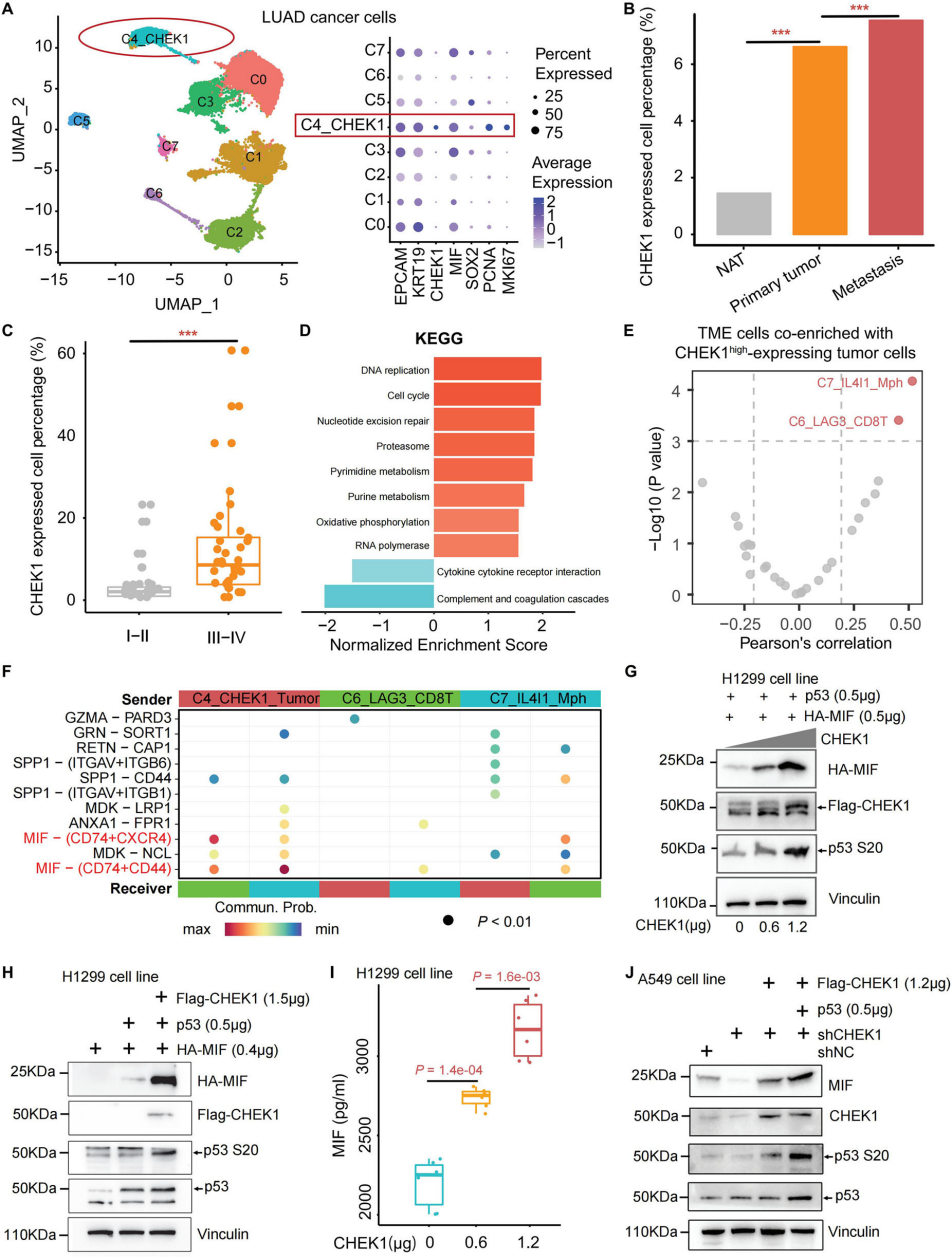
CHEK1 inducing an immunosuppressive microenvironment via MIF signaling to promote LUAD progression. (A) The UMAP plot showed the subpopulations of LUAD cancer cells. *CHEK1* was highly expressed in cluster 4, along with high expression of *MIF*, *SOX2*, *PCNA*, and *MKI67*. (B) Comparison of the percentage of *CHEK1* ^+^ cancer cells in LUAD tumors with corresponding epithelial cells in NATs, and in metastasis tumors, compared to primary tumors using a Chi‐square test. ^***^, *p* < 0.001. (C) Comparison of the percentage of *CHEK1* ^+^ cancer cells in late‐stage (Stage III‐IV) patients to early‐stage (Stage I‐II) patients using a Wilcoxon rank‐sum test. ^***^, *p* < 0.001. (D) GSEA enrichment analysis of the significantly dysregulated KEGG pathways in *CHEK1* ^+^ LUAD cancer cluster. The color represented the normalized enrichment score (NES). (E) The co‐expression patterns of *CHEK1* ^+^ cancer cells with other immune cell populations using Pearson’ correlation analysis. |R| > 0.2 and *p* < 0.001 were deemed to be a significant correlation. (F) Cell‐cell communication between *CHEK1* ^+^ cancer cells and their significantly co‐enriched with C7_IL4I1_Mph and C6_LAG3_CD8T cells. (G) Exogenous MIF expression in H1299 cells co‐transfected with wild‐type p53, in response to graded CHEK1 overexpression (0, 0.6, 1.2 µg). (H) Immunoblot analysis of exogenous MIF, p53, and phosphorylated p53 in H1299 cells overexpressing CHEK1. (I) ELISA quantification of secreted MIF in H1299 cells co‐transfected with wild‐type p53 culture supernatants under increasing *CHEK1* overexpression. Statistical significance was determined by the Wilcoxon rank‐sum test (*ggpubr* R package). (J) Analysis of MIF, p53, and phospho‐p53 expression changes upon rescue of CHEK1‐knockdown A549 cells with exogenous CHEK1 or CHEK1 plus p53.

To assess how functional MIGs shape the TME, we re‐clustered LUAD cancer cells into eight distinct subpopulations (Figure 6A). Among these, the C4 subcluster showed marked enrichment for *CHEK1* (Figure 6A), which was the top‐ranked MIG linked to poor survival in LUAD (Figure 2B). Consistent with our experimental findings (Figure 4G, H) and CRISPR screening (Figure 5B,C), CHEK1‐high cancer cells (C4_CHEK1) showed upregulated expression of proliferation and stemness‐related genes, including *PCNA*, *MKI67*, *SOX2*, and *MIF* (Figure 6A). *CHEK1* expression was significantly higher in tumor tissues compared to NATs and further elevated in metastatic lesions relative to primary tumors, implicating its role in both tumor progression and metastasis (Figure 6B; Table S8). Moreover, the expression of *CHEK1* increased with LUAD progression, showing higher levels in late‐stage compared to early‐stage patients (Figure 6C). Pathway enrichment analysis revealed that CHEK1‐high cancer cells were enriched for oncogenic pathways (e.g., E2F/MYC targets, PI3K/AKT/mTOR, cell cycle) but displayed suppression of immune‐activating pathways (e.g., inflammatory response, cytokine signaling) (Figure 6D; Figure S9).

To investigate the interplay between CHEK1‐high cancer cells and the TME in LUAD, we calculated Pearson correlation coefficients between their abundance and major TME cell populations across patient samples. While pancancer analyses have linked *CHEK1* to FOXP3^+^ Tregs, we found no significant correlation with Tregs in our LUAD cohort. Instead, two distinct immunosuppressive subsets showed strong positive correlations with CHEK1‐high cancer cells: an IL4I1‐high macrophage cluster (C7_IL4I1_Mac) [74] and a LAG3‐high CD8^+^ T cell cluster [61] (C6_LAG3_CD8T) (Figure 6E; Figure S9). This LUAD‐specific pattern directed our subsequent focus to these key cellular interactions. Notably, the C7_IL4I1_Mac subset displayed high PD‐L1 expression (Figure S9E), indicative of its potential to suppress T cell activity. Together, these findings suggest that CHEK1‐high cancer cells contribute to an immunosuppressive TME in LUAD, predominantly through association with PD‐L1^+^ macrophages [75].

To dissect the underlying communication mechanisms, we performed cell–cell interaction analysis among CHEK1‐high cancer cells, IL4I1‐high macrophages, and LAG3‐high T cells. This analysis pinpointed macrophage migration inhibitory factor (MIF) as a pivotal ligand, with the strongest interaction signal emanating from CHEK1‐high cancer cells to IL4I1‐high macrophages, followed by that to LAG3‐high T cells (Figure 6F). Given that CHEK1 phosphorylates p53 at Ser20 [76] and that TP53 co‐occupies active MIF regulatory elements [77] (Figure S10A), we hypothesized that CHEK1 regulates MIF expression via p53 phosphorylation. In H1299 cells co‐transfected with wild‐type p53, increasing doses of CHEK1 induced a dose‐dependent upregulation of MIF (Figure 6G; Figure S10D). To deconvolute the individual contributions of p53 and CHEK1, we performed exogenous expression experiments in p53‐null H1299 cells. Expression of p53 alone only modestly induced HA‐tagged MIF, whereas co‐expression of Flag‐CHEK1 with p53 robustly increased MIF levels (Figure 6H; Figure S10B), indicating that CHEK1 is a key initiator of MIF upregulation in a p53‐dependent manner.

Consistently, ELISA confirmed that CHEK1 overexpression significantly enhanced MIF secretion (*p* < 0.001; Figure 6I). We next established stable CHEK1‐knockdown (shCHEK1) and control (shNC) A549 cell lines with endogenous p53 (Figure 6J; Figure S10C). MIF expression remained unchanged in shNC cells but was markedly reduced upon CHEK1 knockdown. Re‐introduction of CHEK1 into shCHEK1 cells restored MIF expression, concomitant with increased phosphorylation of endogenous p53 at Ser20. Moreover, simultaneous overexpression of CHEK1 and p53 in shCHEK1 cells further enhanced both p53 Ser20 phosphorylation and MIF expression (Figure 6J). Together, these results establish that CHEK1 upregulates MIF expression and secretion through phosphorylation‐dependent activation of p53 at Ser20. Supporting these mechanistic findings, in an independent LUAD cohort (*n* = 56), *CHEK1* and *MIF* expression showed a significant positive correlation in tumors (*n* = 28; linear regression, *p* = 0.023, R = 0.41) but not in matched normal adjacent tissues (*p* = 0.42, R = −0.15) (Figure S9H). Furthermore, *CHEK1* expression was significantly elevated in tumors compared to NATs (Wilcoxon rank‐sum test, *p* = 0.038; Figure S9I), further supporting its role in LUAD pathogenesis.

### 2.7 CHEK1 Shapes an Immunosuppressive Microenvironment via CHEK1‐p53‐MIF Axis

**FIGURE 7:**
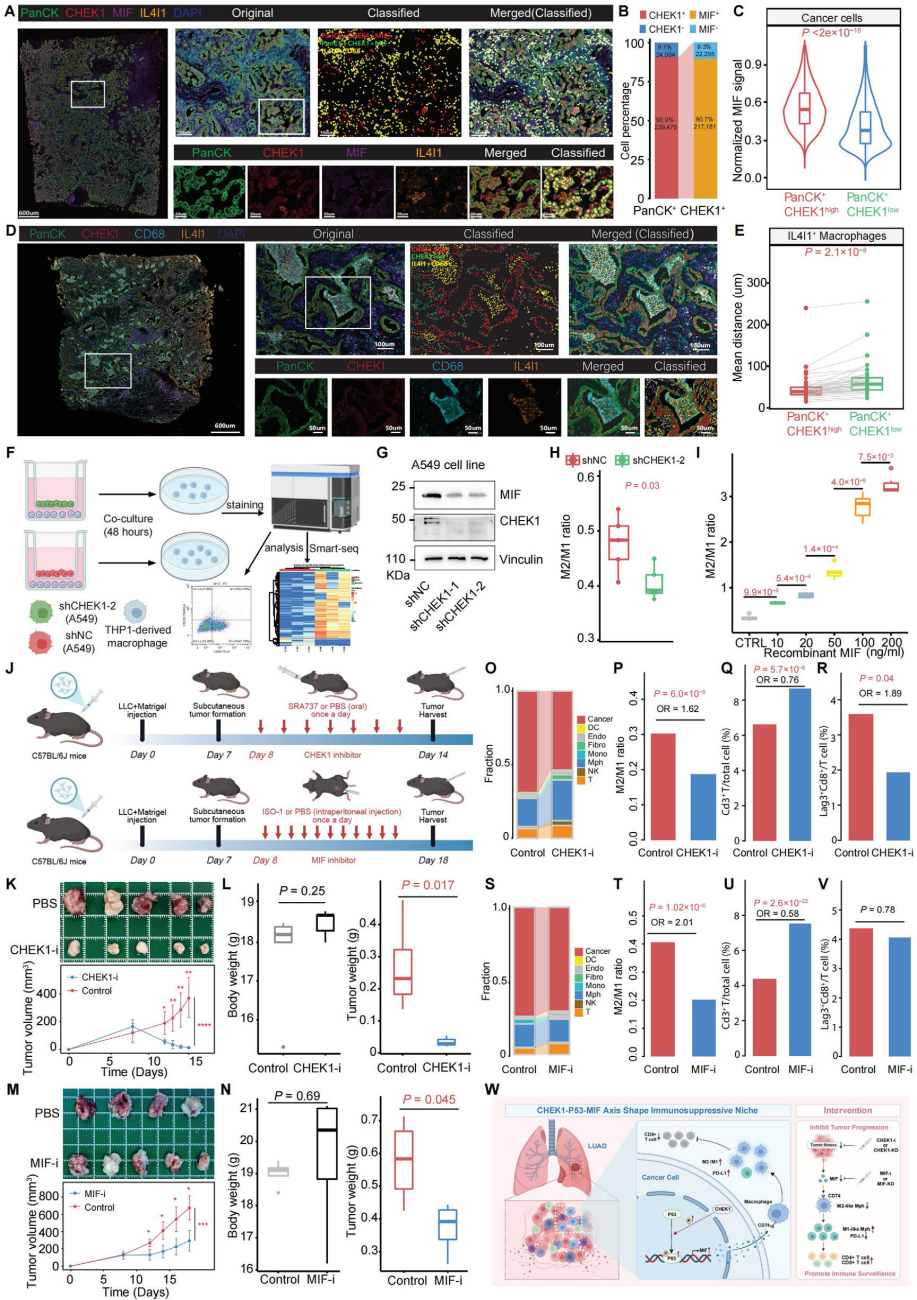
Immunosuppression in LUAD driven by the CHEK1‐MIF axis and rescued by its inhibition. (A) Representative immunofluorescence images of CHEK1 and MIF co‐localization in cancer cells (*n* = 6). (B) Summary of CHEK1^+^ and MIF^+^ cancer cell populations. (C) Comparison of the normalized MIF signal between CHEK1^high^ and CHEK1^low^ cancer cells. (D) Representative immunofluorescence images were shown for mIF of PanCK/CHEK1 (CHEK1^high/low^ cancer cells), and CD68/IL4I1 (IL4I1^+^CD68^+^ macrophages) (*n* = 6). (E) Comparison of the average spatial distances between CHEK1^high^ cancer cells or CHEK1^low^ cancer cells to IL4I1^+^CD68^+^ macrophages (number of ROI = 39). ROI, region of interest. (F) Schematic of the co‐culture workflow. LUAD cells were transduced with non‐targeting (shNTC) or CHEK1‐targeting (shCHEK1) shRNAs prior to co‐culture with THP1‐derived macrophages in Transwell plates for 48h. (G) CHEK1 and MIF protein levels in A549 cells with CHEK1 knockdown. (H) Flow cytometry analysis of macrophage markers (CD206, CD80) following co‐culture with shNTC or shCHEK1 LUAD cells. Statistical analysis was calculated using the two‐tailed *t*‐test. (I) Recombinant human MIF promotes M2‐like polarization of THP‐1 macrophages in a dose‐dependent manner (significance determined by two‐tailed *t*‐test). (J) Experimental timeline for therapeutic intervention post‐subcutaneous LLC inoculation. (K, L) Impact of CHEK1 inhibition (SRA737, 50 mg/kg/day) in a subcutaneous model (*n* = 5/group): tumor volume (K), body weight, and tumor weight (L). (M, N) Impact of MIF inhibition (ISO‐1, 35 mg/kg/day) in a subcutaneous model (PBS, *n* = 4/group): tumor volume (M), body weight, and tumor weight (N). Data are presented as mean ± SEM. Statistical significance was determined by two‐tailed Student's *t*‐test (^*^ *p* < 0.05, ^**^ *p* < 0.01, ^***^ *p* < 0.001, ^****^ *p* < 0.0001). (O–V) CHEK1 or MIF inhibition reprograms the myeloid cells and reinvigorates T cells. (O, S) Stacked bar plot showing the proportional changes of major cell types within tumors treated with PBS (Control), the CHEK1 inhibitor (O) or the MIF inhibitor (S), as defined by scRNA‐seq clustering and annotation. (P, T) M2/M1 macrophage ratio (M2: Cd68^+^Arg1^+^Cd86^−^; M1: Cd68^+^Arg1^−^Cd86^+^) in tumors treated with CHEK1 inhibitor (P) or MIF inhibitor (T) versus PBS. (Q, R, U, V) Percentages of Cd3^+^ T cells (Q, U) and Lag3^+^Cd8^+^ T cells (R, V) in the CHEK1 inhibitor (Q, R) and MIF inhibitor (U, V) groups versus their respective PBS controls. Fisher's exact test was used to determine statistical significance. (W) Mechanism of CHEK1–p53–MIF‐mediated immune evasion in LUAD. In tumor cells, CHEK1‐mediated p53 phosphorylation promotes MIF expression and secretion. Secreted MIF drives macrophage M2 polarization to establish an immunosuppressive microenvironment and promote tumor progression. Intervention of CHEK1 disrupts this axis, impairing tumor‐intrinsic fitness and MIF production, thereby reprogramming macrophages to an immunostimulatory M1‐like phenotype to restore anti‐tumor immunity.

To assess the spatial and protein‐level relationship between CHEK1 and MIF in lung adenocarcinoma (LUAD), multiplex immunofluorescence was employed to visualize key cellular markers within patient tissue sections (Table S9). Robust co‐localization of CHEK1 and MIF was observed within cancer cells (Figure 7A; Figure S13A). Quantification revealed that approximately 91% of cancer cells were CHEK1 positive, and of these, ∼91% were also MIF positive (Figure 7B). Consistent with this, CHEK1‐high cancer cells exhibited significantly greater MIF protein signal intensity compared to their CHEK1‐low cancer cells (Figure 7C). Spatial mapping of the TME revealed that CHEK1‑high cancer cells were strongly co‑enriched with M2‑like macrophages and situated significantly closer to IL4I1^+^ macrophages compared to CHEK1‑low cells (Figure 7D,E; Figure S13B), elucidating the immunosuppressive niche shaped by this axis. A similar spatial association was observed between CHEK1‐high cancer cells and LAG3^+^ exhausted CD8T cells (Figure S11A,B).

Given that MIF drives M2 macrophage polarization and mediates crosstalk between CHEK1‐high cancer cells and M2‐like macrophages [78], we asked whether modulating CHEK1 expression directly regulates macrophage polarization. To test this, we established an indirect co‐culture system (Figure 7F) using PMA‐differentiated THP‐1 macrophages and shCHEK1 tumor cells, which stably exhibit reduced CHEK1 and MIF expression (Figure 7G; Figure S10E). Flow‐cytometric analysis of M1/M2 markers (CD80/CD206) showed that conditioned medium from shCHEK1 cells markedly attenuated M2‐like polarization compared to scramble‐control medium (Figure 7H). This dependency on the CHEK1–MIF axis was further confirmed in an independent LUAD cell line using siCHEK1 and siMIF (Figure S11F–I and S13E,F). Transcriptomic profiling of macrophages isolated from the co‐cultures revealed profound reprogramming upon tumor cell CHEK1 knockdown, with 550 differentially expressed genes in the shCHEK1 group versus shNC controls (Figure S11C,D). GSEA analysis indicated significant activation of anti‐tumor macrophage programs in the shCHEK1 group, including responses to IFN‑γ, IFN‑α, inflammatory signaling, and TNF‑α signaling via NF‑κB [79, 80, 81, 82] (Figure S11E).

To further delineate the functional role of the CHEK1–MIF axis in macrophage polarization, we treated PMA‐differentiated THP‐1 macrophages with recombinant human MIF protein across a concentration gradient (10, 20, 50, 100, and 200 ng/mL). The results confirmed that MIF alone can promote M2‑like polarization in a dose‑dependent manner, consistent with a previous report [78]. More importantly, this finding functionally corroborates our earlier observation that CHEK1‐high cancer cells secrete higher levels of MIF, thereby providing a mechanistic link between CHEK1 upregulation in tumor cells and the subsequent M2‑like polarization in the tumor microenvironment (Figure 7I). Thus, these results reinforce that CHEK1 acts as an upstream regulator of MIF‐mediated macrophage reprogramming, highlighting the CHEK1–MIF–M2 axis as a targetable signaling pathway underlying LUAD immunosuppression.

Having established the CHEK1–MIF–M2 axis as a targetable pathway, we evaluated its therapeutic potential in vivo. Mice bearing subcutaneous lung cancer were treated with either a CHEK1 inhibitor (SRA737 [83]) or an MIF inhibitor (ISO‐1 [84]) (Figure 7J). CHEK1 inhibition significantly suppressed tumor growth, reducing both tumor volume (Figure 7K) and final tumor weight (*p* = 0.017, two‐tailed *t*‐test) with negligible effect on body weight (Figure 7L). MIF inhibition also led to a significant reduction in tumor weight (*p* = 0.045), although this effect was more modest compared to CHEK1 inhibition (Figure 7M,N). Next, we performed scRNA‐seq to dissect the associated immune remodeling and revealed that CHEK1 inhibition reduced tumor cell abundance, increased activated T cells, and broadly reversed immunosuppression (Figure 7O–R; Figure S12A–H). Specifically, it significantly lowered the M2/M1 macrophage ratio (Figure 7P; *p* = 6.0 × 10^−5^, OR = 1.62; Figure S13C), increased activated Cd3^+^ T cells (Figure 7Q; *p* = 5.7 × 10^−8^, OR = 0.76), and reduced the proportions of Lag3^+^Cd8^+^ T cells (Figure 7R; *p* = 0.04, OR = 1.89) and Foxp3^+^Ctla4^+^ T cells (Figure S12E; *p* = 0.03, OR = 1.72; Fisher's exact test). The concomitant reduction of these populations suggests that they may be functionally connected within the immunosuppressive microenvironment modulated by CHEK1 [85, 86]. In contrast, MIF inhibition exerted a narrower immunomodulatory effect (Figure 7S; Figure S12I–L). While it significantly reduced the M2/M1 ratio (Figure 7T; *p* = 1.02 × 10^−6^, OR = 2.01; Figure S13D) and increased activated CD3^+^ T cells (Figure 7U; *p* = 2.6 × 10^−22^, OR = 0.58), it did not alter the proportion of exhausted or regulatory T cell subsets (Figure 7V). This functional disparity may reflect the more rapid plasticity of macrophage polarization compared to the relatively fixed state of T cell exhaustion, as previously suggested [87]. The broader and more coordinated immunomodulation achieved by CHEK1 inhibition underscores its pivotal upstream role in driving immunosuppression across both myeloid and lymphoid compartments.

Furthermore, to evaluate the clinical relevance of CHEK1 in LUAD patients treated with immunotherapy, we analyzed a single‐cell RNA sequencing cohort of 33 individuals who received anti‐PD‐1 therapy [88]. Using a generalized linear model adjusted for age, gender, and smoking history, we found a statistically significant negative association between the proportion of CHEK1‑positive cancer cells and treatment response (*p* = 0.046; Cohen's d = −0.774). The model showed good predictive accuracy with an area under the curve (AUC) of 0.725 (Figure S12M). Consistently, responders harbored significantly fewer CHEK1‑high cancer cells than non‑responders (Figure S12N).

Collectively, our results delineate a CHEK1‐p53‐MIF signaling axis that promotes an immunosuppressive tumor microenvironment in LUAD. We demonstrate that CHEK1 drives MIF secretion, which polarizes macrophages toward a pro‐tumor M2 phenotype. Clinically, CHEK1‐high tumor cells are associated with resistance to anti‐PD‐1 therapy, underscoring the therapeutic potential of targeting CHEK1 to reprogram the immunosuppressive TME and enhance immunotherapy efficacy.

## 3 Discussion

Our study establishes mutation‐intolerant genes (MIGs) as a fundamental class of cancer vulnerabilities that transcend the oncogene‐centric paradigm. By integrating pan‐cancer genomics, single‐cell transcriptomics, CRISPR base‐editing screens with Perturb‐seq readout, and spatial profiling, we demonstrate that MIGs sustain tumorigenesis through dual mechanisms: cell‐intrinsic survival via synthetic lethality and orchestration of an immunosuppressive microenvironment.

Moving beyond single‑gene saturation mutagenesis studies (e.g., *BAP1* [1], *BRCA2* [4, 5]), our work establishes a systematic framework that leverages natural genetic variation from normal tissues to identify mutation‑intolerant genes (MIGs)—genes tolerated in healthy cells but essential in cancer, offering a therapeutically attractive strategy with high clinical potential. Perturb‑seq provided direct causal evidence that MIG editing downregulates their own expression and disrupts oncogenic pathways, pinpointing the mechanistic basis of this vulnerability. Focusing on the top‑ranked MIG, *CHEK1*, we found that both shRNA knockdown and CRISPR base‑editing consistently suppressed LUAD proliferation, confirming its role as a synthetic‑lethal vulnerability. We identified a distinct CHEK1‑high cancer cell subpopulation with stem‑like properties (elevated *SOX2* [66] and *MIF* [63]). Within this subpopulation, CHEK1 activates multiple pro‑tumor pathways (E2F, MYC, PI3K/AKT/mTOR) known to drive progression [70, 89] and modulate communication [90].

Correlation and spatial analysis revealed that CHEK1‑high cells positively associate with immunosuppressive IL4I1^+^ macrophages and LAG3^+^ CD8^+^ T cells. Cell‑cell communication analysis indicated these interactions are mediated via the MIF‑CD74 axis. Functionally, *CHEK1* overexpression upregulates *MIF* expression and secretion in a p53‑Ser20 phosphorylation‐dependent manner. Conversely, CHEK1 inhibition attenuated MIF and shifted macrophages toward an immunostimulatory M1‑like phenotype through IFN/NF‑κB signaling [79, 80, 81, 82]. Collectively, these results establish CHEK1 as a non‐oncogene vulnerability that exhibits two defining and complementary features: strong negative selection against mutations alongside transcriptional upregulation within an aggressive tumor subpopulation. This duality reflects underlying biological constraints. Mutational intolerance indicates that loss‐of‐function variants are eliminated because CHEK1 activity is indispensable for tumor fitness. In parallel, elevated expression of the wild‐type CHEK1 allele signifies a co‐opted transcriptional program that promotes stemness, mitigates replication stress, and, as we show here, directs immune evasion through the p53‐MIF axis. CHEK1, therefore represents a targetable dependency in which the wild‐type protein is commandeered to sustain malignancy.

In vivo, targeting either CHEK1 or MIF remodeled the TME, with CHEK1 inhibition exerting a broader immunomodulatory effect, underscoring its role as an upstream regulator. Critically, high tumor *CHEK1* expression predicted poorer response to anti‑PD‑1 therapy in an independent cohort, linking this axis directly to clinical immunotherapy resistance. This aligns with prior evidence linking CHEK1 overexpression to immunosuppressive phenotypes and unfavorable outcomes [91]. To fully realize this therapeutic potential, future studies could integrate advanced multidimensional profiling and analytical frameworks, such as those combining multi‐omics, visualization, and artificial intelligence as applied in next‐generation cell therapies [92]. This would enable a systematic mapping of the tumor‐immune interactions modulated by CHEK1 and accelerate the identification of rational combination strategies with immune checkpoint blockade. Moreover, CHEK1 acts as an upstream regulator of MIF, positioning this axis as a promising therapeutic target for both cancer immunotherapy and other cytokine‐driven pathologies [93]. Therefore, targeting CHEK1 represents a promising strategy to reprogram the immunosuppressive TME and enhance immunotherapy efficacy. While this study delineates the prototypical CHEK1‑p53‑MIF‑CD74 axis, the molecular functions of the broader MIG repertoire present a rich landscape for future exploration.

## 4 Conclusion

Our study establishes mutation‐intolerant genes (MIGs) as a previously overlooked class of cancer vulnerabilities that function independently of the traditional oncogene‐tumor suppressor paradigm. Through pancancer fine‐mapping and multi‐omics integration analysis, we demonstrate that MIGs sustain tumor progression via two mechanisms: maintaining tumor intrinsic fitness and promoting immune evasion. Strikingly, we delineate a CHEK1–p53–MIF–CD74 axis in LUAD that supports tumor‐intrinsic fitness and drives M2‐like macrophage polarization, thereby shaping an immunosuppressive niche. Targeting this axis remarkably reverses immunosuppression, and high CHEK1 expression is associated with resistance to anti PD 1 therapy, highlighting the potential of MIGs for tumor‐specific treatments with minimal toxicity to normal tissues.

## 5 Experimental Section

### 5.1 Ethical Statement

The research adhered to the Declaration of Helsinki and was ethically approved by the Ethics Committee of the Zibo Central Hospital (No. R2024‐060).

### 5.2 Omics Data Pre‐Processing

To identify and prioritize mutation‐intolerant genes (MIG) in tumor genomes, we gathered somatic mutations from both cancer tissues and healthy human tissues, mRNA data from cancer tissues, clinical information of cancer patients, and cancer cell vulnerabilities. The somatic mutations from 8994 healthy samples in 25 human tissue types and corresponding sample information retrieved from the SomaMutDB [27]. The pan‐cancer somatic mutations from 10 769 tumor samples in 33 cancer types, generated by the multi‐center mutation‐calling in multiple cancers (MC3) network [26]. Similar to our previous studies [94, 95], ANNOVAR [96] was used for comprehensive annotation of all somatic mutations at the gene‐region levels (exonic, intronic, UTR5, UTR3, etc.) and the variant‐levels (frameshift, nonframeshift, synonymous, nonsynonymous, stopgain, stoploss, etc). We retained cancer types across TCGA tumors had corresponding healthy tissue types for downstream analysis. In total, 8096 tumor samples from 23 cancer types and 6603 healthy samples from 21 human tissues were retained (Figure S1A,B). The vulnerabilities of cancer cells (gene‐dependence scores from 1096 models of 150 model types) [97], gene‐drug interaction data [98], and the transcripts per million mapped reads (TPM) value from the tumor and NAT sample in LUAD [99], were used for integration analysis.

### 5.3 Identification of Significantly Mutated Genes

MutSigCV [100] (v. 1.41) was employed to identify the significantly mutated genes (SMGs) in both healthy control and cancer tissues. Genes with a false discovery rate (FDR) < 0.1 were considered as SMGs (Figure S1A).

### 5.4 Identification of Mutation‐Intolerant Genes in Tumors

We developed a computational procedure, termed mutation intolerant driver (miDriver), to identify the MIGs in tumors by comparing them with anatomically matched healthy tissues (Figure 1A). For each of the tissue‐matched tumor–healthy pairs, somatic mutations were separately analyzed with MutSigCV [100] to evaluate gene‐level mutation tolerance. Mutation tolerance in matched healthy tissues was quantified as *P*, while mutation intolerance in cancer tissues was correspondingly defined as 1‐*P*. Owing to limited sample size, any healthy or tumor tissue type that could not be reliably evaluated by MutSigCV was excluded from downstream analysis. The two matched *p*‐values for each gene from the remaining 15 tumor‐healthy tissue pairs were combined using Fisher's method to produce an overall significance metric. The combined *p*‐value for each gene in each cancer type was calculated as follows:

(1) $$Combined\;P=-2\sum_{i}\ln\left(P_{i}\right)∼\chi_{6}^{2}$$

Here, *i* represents mutation tolerance in healthy tissue and mutation intolerance in cancer tissue. The combined *p*‐values were adjusted using the FDR. Furthermore, cancer cell vulnerability data were integrated to prioritize the candidate genes. Ultimately, 1020 MIGs were identified from 13 tumor‐healthy tissue pairs with vulnerability data in corresponding cancer cell lines, with a FDR < 0.1 and a mean CRISPR gene‐dependence score > 0.9. The top 30 mutation‐intolerant genes (MIGs) were selected using a multi‐step approach designed to identify genes showing significantly different mutational patterns between matched healthy and cancer tissues. Selection was based on three integrated criteria: (1) mutation intolerance in cancer, defined by an FDR > 0.6 from MutSigCV, indicating lack of significant mutational enrichment in tumors; (2) significant mutation tolerance in corresponding healthy tissues (FDR < 0.1); and (3) quantitative validation through a log2‐transformed non‐silent mutation burden ratio (healthy control/corresponding cancer) > 1. This combined approach ensures the identification of genes under strong negative selection in cancer environments.

### 5.5 Enrichment and Survival Analysis

Metascape [101] was utilized for biological pathway enrichment analysis with MIGs. A q value lower than 0.05 was employed for the selection of significantly enriched biological pathways. Gene Set Enrichment Analysis (GSEA) was conducted using gene activity within the module based on gene sets from the MSigDB Hallmark [102]. Significantly enriched hallmarks were identified with an FDR < 0.05. The R package survival (v. 3.5–8) was utilized to calculate the survival probability of each patient. Survival‐related MIGs were defined with a *p* < 0.05. Survival curves were generated using the *survminer* package (v. 3.5–8) with the ggsurvplot function.

### 5.6 Prognostic Model

The CMPS prognostic score was derived from cell cycle‐related MIGs, which were defined as those significantly enriched in three core cell cycle pathways from the MSigDB database: GO:0000278 (mitotic cell cycle), GO:0010564 (regulation of cell cycle process), and hsa04110 (Cell cycle). To account for inherent differences across cancer types, we applied LASSO Cox regression analysis [35] independently to each tumor type. This approach utilized expression data of the cell cycle MIGs in each specific cancer context, employing 10‐fold cross‐validation with optimization to the minimal λ value for robust gene selection and prognostic model construction. The diagnostic accuracy of the constructed model based on CellCycle MIGs was assessed via time‐dependent area under the receiver operating characteristic (ROC) curve (AUC) and the concordance index (C‐index). ROC curves and AUC value were generated using the *risksetROC* package (v. 1.0.4.1) [103].

The coefficients and expressions of the selected CellCycle MIGs in the constructed model were integrated to calculate the CellCycle MIG‐related prognostic risk score (CMPS) for each patient in the corresponding cancer type, calculated as follows:

(2) $$CMPS_{i,t}=\sum_{g}\mathrm{Coefficient}\;\left(gene_{g,t}\right)\times \mathrm{Expression}\left(gene_{i,g,t}\right)$$

where *g* stands for each of the final selected CellCycle MIGs, *t* stands for each cancer type, and *i* stands for each individual patient.

Additionally, the prognostic significance of MIGs was also evaluated in independent cohorts. Briefly, patients in each cancer cohort were divided into a 70% training set and a remaining 30% testing set. The diagnostic accuracy was estimated in the 30% testing set. The median value of CMPS was utilized to group patients into high or low‐risk categories in each cancer type for survival analysis.

### 5.7 Analysis of Immune Cell Infiltration Using Deconvolution Methods

The immune cell infiltration in tumors of each cancer was assessed using CIBERSORTx [104] (https://cibersortx.stanford.edu/) with batch effects normalized mRNA data. The Wilcoxon‐rank sum test was applied to compare the abundance of each type of immune cell between the high and low groups of CMPS. The Spearman correlation test was utilized to assess the association between the abundance of each type of immune cell and CMPS scores. The *p*‐values were adjusted using FDR. A significance threshold of FDR < 0.05 was applied in this analysis.

### 5.8 Pan‐Cancer scRNA‐seq Integration Analysis

In addition, we collected fifteen publicly available scRNA‐seq cancer datasets containing either raw FASTQ or processed matrix files for integration analysis. Raw FASTQ data were aligned to the human reference genome (hg38) and quantified using Cell Ranger (v. 7.1, 10x Genomics Inc). To obtain high‐quality data, cells from each sample that passed all the following filters using Seurat (v. 4.4.0) [105] were retained for downstream analysis: (1) with > 200 genes expressed; (2) with > 500 counts detected; (3) with < 15% counts in mitochondrial genes detected; and (4) classified as ‘Singlet’ using DoubletFinder with default settings. Subsequently, the datasets were merged according to cancer type. Finally, we acquired six meta scRNA‐seq cohorts, including 280 samples and 874 132 cells (Table S4).

For the identification of major cell populations, the six meta datasets were first pre‐processed separately as follows: (1) normalized using the NormalizeData function with default settings; (2) selected the top 3000 features using the FindVariableFeatures function with the parameter ‘d*ispersion.function = LogVMR*’; (3) scaled using the ScaleData function with default settings; (4) reduced dimension using the RunPCA function with the parameter ‘*npcs = 30*’; (5) removed batch effects using Harmony [106]; (6) further reduced dimension using Harmony and identified nearest neighbors. The pre‐processed dataset for each cancer type was then used for cell cluster identification with a resolution of 0.8. The well‐known marker genes include: *CD3D*, *CD3E*, *NKG7*, and *GNLY* for T and NK cells; *CD79A*, *MS4A1*, and *MZB1* for B and plasma cells; *CD14* and *CD68* for the myeloid lineage; *COL1A2* and *FAP* for fibroblasts; *GLDN5* and *VWF* for endothelial cells; *MS4A2* and *TOP2A* for mast and cycling cells; and *EPCAM*, *KRT19* and *ALDH1A1* for epithelial cells. For epithelial cells, cells originating from tumor samples were considered as cancer cells, while those from non‐adjusted tumor (NAT) samples were considered as normal epithelial cells. The major cell populations, including B cells, T and NK cells, and myeloid cells, were merged from the six cancer types for further sub‐population characterization with a resolution of 0.2. According to CellMarker (v. 2.0) [107], sub‐clustering marker genes included: *CD44* and *TXNIP* for memory B cells (MB); *MZB1* for activated memory B cells (AMB); *TCL1A* for naive B cells (NB); *CD8A*, *CD8B*, *GZMA*, *GZMB*, *GZMM*, *GZMH*, GZMK, and *NKG7* for cytotoxic CD8 T cells (CTL); *GNLY* for natural killer cells (NK); *CD4*, *CTLA4* and *FOXP3* for regulatory T cells (Treg); *IL7R* for naïve T cells; *CD14*, *CD163*, *APOE*, *C1QA*, and *C1QC* for macrophages (Mph); *S100A9* and *S100A8* for monocytes (Mono); *CLEC9A*, *XCR1*, and *BATF3* for type 1 dendritic cells (DC1); *CD1C*, *CD1E*, and *CLEC10A* for type 2 dendritic cells (DC2); *CCR7*, *LAMP3*, *FSCN1*, and *CCL22* for type 3 dendritic cells (DC3); *CCR7*, *LAMP3*, FSCN1 and *CCL22* for type 3 dendritic cells (DC3); *LILRA4*, *IL3RA*, and *TCF4* for plasmacytoid dendritic cells (pDC); *TOP2A* for proliferating myeloid cell (pMye); For macrophage cells, we further evaluated their M1/M2 polarization ability using the AddModuleScore function (Figure S4I, Table S4).

To validate the contribution of MIGs to tumor progression at the single‐cell level, we used the prognostic MIGs that positively contributed to patient survival, as identified in the ‘Prognostic model’ section (Table S6), to estimate their expression in the epithelial or cancer cells of each cancer, respectively. Cells with more than seven read counts detected were defined as expressed. The significance for the ratio of expressed cells between the primary tumor and NAT, metastasis tumor and NAT, tumor (primary tumor and metastasis tumor) and NAT, and metastasis tumor was evaluated using the ‘rateratio.test’ function as utilized in our previous studies [108, 109] (Figure 3B). The FindAllMarkers function in Seurat was utilized to identify genes significantly higher expressed in cancer cells expressing prognostic MIGs compared to the rest cancer cells for each cancer type (Figure 3C). The Fgsea (fast GSEA) R package was employed for enrichment analysis at the single‐cell level based on a pre‐ranked gene list of “avg_log2FC” from the results of FindAllMarkers. Adjusted *p*‐values lower than 0.05 were considered as significant ones (Figures 3D, 6D; Figure S9F,G). To further investigate the influence of cancer cells expressing prognostic MIGs on TME, we evaluated the association between the fraction of cancer cells expressing prognostic MIG and the fraction of each subcluster of immune cells in the corresponding tumor samples using Pearson correlation coefficient analysis (Figure 3E). The cell‐cell communications between the interested cancer cells and TME cells were evaluated using CellChat (v. 1.6.1) [110] following the default pipeline (Figures 3F and 6F).

To further explore the mechanism of prognostic MIGs in LUAD tumor progression, we re‐subclustered the cancer cells, B cells, T cell, NK cells, and myeloid cells in the LUAD cohort with a resolution of 0.2 (Figure 6A; Figure S9A–C). The same marker genes used in Pan‐cancer were employed for LUAD cell‐type annotation. For macrophage cells, immune suppression and M2 macrophage signatures were collected from a previously published LUAD cohort [111]. The immune suppression scores and M2 signature scores were evaluated using the AddModuleScore function (Figure S9D,E).

### 5.9 Cell Lines

A549 (Li Kailong Laboratory) cells were maintained in RPMI‐1640, H1299 (Cell Resource Center, CAMS), HEK293T (Li Kailong Laboratory), and mouse LLC (Procell, CL‐0140) cells were cultured in DMEM. All media (Gibco) were supplemented with 10% FBS (Thermo Fisher Scientific) and 1% penicillin‐streptomycin. All cells were incubated at 37°C with 5% CO_2_ and confirmed to be mycoplasma‐free using a detection kit (InvivoGen, rep‐mys‐10).

### 5.10 Integrated Single‐Cell and Bulk CRISPR Base‐Editing Screening

A pooled single‐guide RNA (sgRNA) library was designed to target interesting mutation sites in MIGs, which mutations occurred in healthy people while absent in corresponding tumor tissue. For each site, we designed 1–3 sgRNAs. All guides were selected to target within the optimal activity window for the respective base editor: positions 4–8 (counting from the PAM‐distal end) for adenine base editing (ABEmax‐NG) and positions 3–7 for cytosine base editing (Anc‐BE4max‐NG). An oligonucleotide pool encoding the sgRNA sequences, flanked by constant regions for cloning into the lentiguide‐MS2‐Puro backbone (Addgene #133739), was synthesized (Twist Bioscience). The library was cloned via Golden Gate assembly, transformed into Endura electrocompetent cells (Lucigen) to achieve >500x coverage, and maxi‐prepped for viral production.

Lentivirus encoding the sgRNA library were generated with the sgRNA library plasmid, pMD2.G, and psPAX2. Then, HEK293 and H1299 cells were infected at a low multiplicity of infection (MOI < 0.3) to minimize multiple sgRNA integrations. Cells were selected with BFP (Blue Fluorescent Protein) for 3 days to generate a stable polyclonal pool. Library representation was confirmed by deep sequencing of the integrated sgRNA repertoire from genomic DNA.

To capture both the initial state and the phenotypic consequences of base editing, the stable H1299‐sgRNA pool was divided at the point of BFP selection. The Day 0 aliquot was processed directly for bulk gDNA extraction (DNeasy Blood & Tissue Kit, Qiagen). In parallel, the Day 3 aliquot was transfected with the respective base editor (ABEmax‐NG or Anc‐BE4max‐NG) and then subjected to Perturb‐seq after a 3‐day outgrowth period. For Single‐Cell Perturb‐seq, 1.2 × 10^5^ cells were loaded onto a 10x Genomics Chromium Controller using the Chromium Next GEM Single Cell 5' Kit v2 (10x Genomics, PN‐1000263), strictly following the manufacturer's protocol.

Libraries were constructed according to the standard protocol for the 10x Genomics Single Cell 5' Solution. The resulting Gene Expression Library and the Feature Barcode Library (containing the sgRNA sequences) were quantified, pooled at an appropriate molar ratio, and sequenced on an Illumina platform. A sequencing depth of ≥ 50 000 reads per cell was targeted for the gene expression library, and the feature barcode library was sequenced to a high depth (> 5,000 reads per cell) to ensure confident sgRNA identification. The Bulk sgRNA amplicon libraries were sequenced on an MGI DNBSEQ‐T7 platform using a 150 bp paired‐end run to a minimum depth of 100x coverage per sgRNA. The sgRNA abundances were quantified from the fastq files using the MAGeCK (version 0.5.9.5) count function.

Cell Ranger (v7.1.0) was used to perform sample demultiplexing, barcode processing, and alignment of mRNA reads to the self‐constructed reference genome with edited sites and 200bp flanking sequence. The Feature Barcode analysis pipeline in Cell Ranger was used to count sgRNA UMIs per cell barcode, using a custom CSV file containing the library's sgRNA sequences. Downstream analysis was performed in R using the Seurat (v4.3.0) package. Cell barcodes were rigorously filtered to retain only high‐quality cells: number of unique genes > 500, total UMIs > 300, and mitochondrial percentage < 10%. Cells were further filtered to include only those with exactly one sgRNA assigned, ensuring unambiguous genotype‐phenotype linkage. The gene expression matrix was normalized using SCTransform and integrated. Dimensionality reduction was performed via PCA and UMAP. Differential gene expression analysis for each perturbation was performed by comparing cells containing a specific sgRNA (or aggregate of sgRNAs for the same gene) to cells expressing non‐targeting control sgRNAs. Gene Set Enrichment Analysis (GSEA) was performed on pre‐ranked gene lists to identify significantly enriched or depleted biological pathways.

### 5.11 Phenotype Assessment Using GLM Model

We employed a generalized linear modeling framework with likelihood ratio testing to quantify the phenotypic impact of each gRNA perturbation. The model was defined as: logit(p) = β_0_ + β_1_ × I_gRNA + β_2_ × F_D0, where p is the probability of a cell containing the gRNA, I_gRNA is a binary indicator for the gRNA (1 for target gRNA, 0 otherwise), and F_D0 is its normalized gRNA frequency at Day 0. The coefficient β_1_ represents the gRNA's effect size. For intuitive interpretation, we converted β_1_ to a log2 fold‐change (log2FC) value, where a positive log2FC indicates enhanced cellular proliferation, and a negative log2FC indicates impaired proliferation/ lethal. Then, all tested gRNAs were adjusted for multiple testing using the Benjamini–Hochberg method, with a significance threshold of FDR < 0.05.

### 5.12 Permutation Test and PPI Network Analysis

To assess co‐expression networks between mutation‐induced genes (MIGs) and established LUAD drivers, we performed permutation tests using RNA‐seq data from LUAD tumors. Known LUAD drivers were curated from the OncoVar database [112], and co‐expression relationships were analyzed as follows: (1) Pairwise correlation analysis: Pearson correlations were computed between all protein‐coding gene pairs using LUAD tumor transcriptomes. Co‐expressed pairs were defined as those with absolute correlation coefficients (|R|) > 0.5 and *p* < 1.0 × 10^−8^, following our prior framework [108, 109]. (2) Permutation‐based significance: Co‐expression frequencies between 267 LUAD‐specific MIGs and LUAD drivers were compared against 1 000 000 iterations of randomly selected gene sets matched for size (*n* = 267) and expression variance. Similarly, to evaluate pan‐cancer MIG enrichment in synthetic lethal (SL) interactions, we applied permutation testing (1 000 000 iterations) comparing 1020 pan‐cancer MIGs against size‐matched random gene sets (*n* = 1020), using published SL pairs (Fielden et al., 2025 [10]) as the reference. The PPI network was constructed and analyzed using Cytoscape (version 3.10.1), where node attributes were mapped to degree centrality values to identify potential hub proteins within the interaction network.

### 5.13 Gene Knockdown Using shRNA and siRNA

To ensure robust validation of our findings, parallel experiments were performed in two human non‐small cell lung cancer cell lines, A549 and H1299, using both short‐hairpin RNA (shRNA) and small interfering RNA (siRNA)‐mediated knockdown approaches. A549 and H1299 cells were cultured in Dulbecco's modified Eagle's medium (HyClone, SH30022) supplemented with 10% fetal bovine serum, 50 U/mL penicillin, and 50 µg/mL streptomycin, at 37°C in a humidified 5% CO_2_ incubator. All cells were checked for mycoplasma using the TransDetect PCR Mycoplasma Detection Kit (TRANGENE, FM311‐01). shRNA sequences targeting the gene of interest were designed using the Broad Institute's online tool and cloned into the pLKO.1 vector. siRNA sequences were designed and synthesized by GeneChem. Transient transfections were performed utilizing the Lipofectamine 3000 reagent (Thermo Fisher Scientific, Cat# L3000015) in line with the manufacturer's established instructions. Cells transfected with a non‐targeting shRNA vector or a negative control siRNA served as the respective controls (Table S5).

### 5.14 Verification of Knockdown Efficiency

Knockdown efficiency was evaluated by quantitative real‐time PCR (qRT‐PCR) 48 h after transfection. For shRNA‐mediated knockdown in A549 cells, total RNA was extracted using MagZol reagent (Magen, R4801‐01) and reverse‐transcribed with the HiScript 1st Strand cDNA Synthesis Kit (Vazyme, R312‐01). qRT‐PCR was performed on a QuantStudio 3 instrument using Universal SYBR qPCR Mix (Magen, MD70101S). Gene‐specific primers and GAPDH primers (designed and validated via NCBI) were used for amplification. For siRNA‐mediated knockdown in H1299 cells, total RNA was isolated with the FastPure Cell/Tissue Total RNA Isolation Kit V2 (Vazyme, RC112‐01) and reverse‐transcribed with the HiScript III 1st Strand cDNA Synthesis Kit (+gDNA wiper) (Vazyme, R312‐02). qRT‐PCR was performed on QuantStudio 3 instrument using Taq Pro Universal SYBR qPCR Master Mix (Vazyme, Q712‐02). Primers specific for MIF and GAPDH were purchased from Beyotime Biotechnology. Primers specific for CHEK1 were designed and validated via NCBI.

### 5.15 Wound Healing Assay

The wound healing assay was performed to evaluate cell migration. A549 cells were inoculated into 6‐well plates at an optimized density and maintained under standard incubation conditions until achieving a confluent monolayer (90%–100% confluence). A straight scratch was created in the cell monolayer using a sterile 200 µL pipette tip. The wells were gently washed with PBS to eliminate detached cells and debris, and fresh medium supplemented with no FBS was added. Images of the wound area were captured at 0, 24, and 48 h using an Olympus IX73 microscope. The wound width was measured using ImageJ software (NIH, USA), and the wound closure percentage was calculated using the following formula:

(3) $$\begin{array}{ccc} & & \mathrm{Wound}\;\mathrm{Closure}\;\mathrm{Percentage}\\ & & =\frac{Initial\;Wound\;Area-Remaining\;Wound\;Area\;at\;Time\;Point}{Initial\;Wound\;Area}\\ & & \times 100\% \end{array}$$

### 5.16 CCK‐8 Assay

Cell viability and proliferation were evaluated using the Cell Counting Kit‐8 (CCK‐8) assay (Lablead, CK001). A549 cells were seeded in 4 independent 96‐well plates at a density of 3000 cells per well. After 0, 24, and 48 h, 10 µL of CCK8 solution was added to each well, and the plates were incubated for an additional 1.5 h at 37°C. The absorbance was measured at 450 nm using an iMark microplate reader (Bio‐Rad, USA). The cell viability was calculated relative to the control group, and data was presented as mean ± standard deviation (SD) of 6 replicates (wells) at each time point.

### 5.17 RNA‐seq Analysis for LUAD Cell Line

Total RNA was isolated from LUAD A549 cancer cells with each candidate gene knockdown and control cells by using MagZol reagent (Magen, R4801‐01) following the manufacturer's protocol. The mRNA libraries for DHX37 knockdown and control cells were generated using the Hieff NGS MaxUpTM II Dual‐mode mRNA Library Prep Kit (YEASEN, 12300ES08) for Illumina and sequenced on Illumina NovaSeq X Plus (paired‐end 150 cycles). For CHEK1 and CDC45 knockdown and control cells, mRNA libraries were constructed according to the operating instructions of VAHTS Universal V6 RNA‐Seq Library prep kit for Illumina (NR 604‐01/02). Subsequently, DNBSEQ‐T7 (150bp paired‐end) was employed for RNA sequencing. Fastp [113] (v. 0.12.4) was used to process raw RNAs‐seq FASTQ data. After quality control, the processed reads were aligned to the human reference genome (hg38) using hisat2 [114] (v. 2.2.1). Over 88% of reads from each candidate gene knockdown and control sample uniquely mapped to the reference genome. The uniquely mapped reads were aggregated into gene‐level counts utilizing featureCounts [115] (v2.0.1) with GENCODE gene annotation (v38) [116]. Differentially expressed genes (DEGs) were then identified using DESeq2 [117] (v. 1.38.3) between control A549 cells and each gene knockdown A549 cells. Genes with fold change ≥1.5 and FDR < 0.05 were determined as significantly differentially expressed genes.

### 5.18 Western Blotting Assay

The Flag‐CHEK1 and HA‐MIF plasmids were constructed using the pCDH‐CMV‐MCS‐EF1‐Puro backbone. H1299 cells were co‐transfected with HA‐MIF plasmids (0.5 µg) without Flag‐CHEK1 plasmid, with 0.6 and 1.2 µg Flag‐CHEK1 plasmid were collected and lysed in RIPA buffer (20 mm Tris‐HCl, pH 7.5, 150 mm NaCl, 1 mm EDTA, 2 mm Na3VO4, 5 mm NaF, 1% Triton X‐100) with a Protease inhibitor cocktail (Solarbio, IKM1010) and eluted by SDS loading buffer. The proteins were resolved in 15% SDS‐PAGE electrophoresis and transferred to PVDF membranes. Blots were incubated with primary antibodies (Anti‐Flag ab 1:1000 F9291 Sigma–Aldrich Anti‐HA ab 1:1000 11867423001 ROCHE) overnight at 4°C followed by HRP‐conjugated species‐specific antibodies (ZB‐2301, ZB‐2305). The results were obtained with the ECL Reagent enhanced chemiluminescence (ECL) reagent (P1050‐100, Applygen) after incubation with appropriate primary and secondary antibodies as indicated. The protein expression of MIF and CHEK1 were also evaluated in shCHEK1 and shNC A549 cells. To evaluate whether the expression of MIF is regulated by TP53 phosphorylation in tumor cells, H1299 cells were co‐transfected with 0.5 µg HA‐MIF plasmid and empty vector and 0.6 and 1.2 µg Flag‐CHEK1 plasmid. The following primary antibodies were used for western blotting in this study: anti‐phospho‐CHEK1 (Cell Signaling Technology, 2341T, 1:1000), anti‐p53 (Cell Signaling Technology, 13684, 1:1000), and anti‐phospho‐p53 (Ser20) (Abcam, ab157454). To validate these observations under endogenous p53 conditions, the constructed stable CHEK1‐knockdown (shCHEK1) and negative control (shNC) A549 cell lines were lysed, and protein extracts were subjected to western blotting to assess the levels of MIF, CHEK1, phosphorylated p53 (Ser20), and total p53. GAPDH or β‐actin served as the loading control.

### 5.19 ELISA for Secreted MIF Analysis

Conditioned media were collected 48h after transfecting H1299 cells (*n* = 6 biological replicates) with 0.5 µg HA‐MIF, either without or with 0.6 or 1.2 µg Flag‐CHEK1 plasmid. Secreted MIF levels were quantified using the human MIF Picokine ELISA kit (Boster, EK0813) according to the manufacturer's protocol. Absorbance was measured at 450 with 540 nm reference on a BioTek Synergy H1 plate reader, with concentrations calculated against a 4‐parameter logistic standard curve (0–2000 pg/mL).

### 5.20 Multiplex Immunofluorescence

To spatially validate the role of the CHEK1‐MIF axis in remodeling the immunosuppressive tumor microenvironment, we conducted multiplex immunofluorescence (mIF) on FFPE tumor samples from 12 LUAD patients (Table S9) using the Opal 6‐Plex Manual Detection Kit (AKOYA BIOSCIENCES). First, we assessed the co‐localization of CHEK1 and MIF using a panel of antibodies against PanCK, CHEK1, and MIF. Subsequently, to map the spatial context of CHEK1‐high tumor cells relative to key immunosuppressive subsets (LAG3^+^CD8^+^ T cells and IL4I1^+^ M2‐like macrophages), we performed a second staining using antibodies against PanCK, CHEK1, LAG3, CD8, CD68, and IL4I1 (all antibody details are listed in Table S9). Following complete staining, slides were counterstained with DAPI and scanned using the Akoya Bioscience inForm platform (v. 3.0).

For image analysis, the scanned images were uploaded to the software interface for manual designation of regions of interest (ROIs) within the tissue sections. For each individual image, we identified an average of nine areas enriched with tumor cells and their corresponding stained cells. Within these ROIs, single‐cell segmentation was performed by the machine learning algorithms of inForm 3.0, based on nuclear, cytoplasmic, and membrane markers. To define tumor cell phenotypes, we classified CHEK1‐high cancer cells as PanCK^+^ epithelial cells with a mean cytoplasmic CHEK1 intensity > 0.4, and CHEK1‐low cancer cells as those with an intensity ranging from 0.1 to 0.4. Based on this classification, we then examined their spatial distribution and quantified the spatial distances between CHEK1‐high / CHEK1‐low cancer cells and the immune cell populations within a 400µm radius in the chosen ROIs.

### 5.21 Co‐Culture of shCHEK1 LUAD Cells With THP‐1‐Derived Macrophages

For co‐culture preparation, THP1‐T676c3 were purchased from the National Stem Cell Translation and Innovation‐Biomedical Cell Resource. The ready shNC and shCHEK1 cells were incubated in fresh DMEM medium containing 10% FBS. THP‐1 monocytic cells were maintained in RPMI‐1640 medium supplied with 10 mm beta‐ME.

The co‐culture system was established using a 6‐well Transwell system (0.4 µm pore size; Corning). THP‐1 macrophages (6 × 10^5^ cells/well) were seeded in the lower chamber, and differentiated into macrophages by treatment with 50 ng/mL phorbol 12‐myristate‐13‐acetate (PMA) for 72 h in RPMI‐1640 medium supplemented with 10% FBS in the lower chamber of a 6‐well plate. At this point, the cells are M0. For comparison, the M0 group is continuously cultured with a final concentration of 100 ng/mL LPS and 20 ng/mL IFN‐γ and treated for 48 h to induce M1 macrophages. M0 cells are treated with a final concentration of 20 mg/mL IL‐4 and 20 mg/mL IL‐13 for 48 h to induce M2 macrophages. shNC and shCHEK1 A549 cells (4 × 10^5^ cells/well) were placed in the upper chamber, seeded the day before co‐culture. The co‐culture was maintained in RPMI 1640 medium at 37°C in 5% CO_2_ for 48 h. After co‐culture, the lower cells were harvested via Accutase (Biolegend, 423201) and stained with CD80 PE (305207) and CD206 BV421 (321125) (Biolegend) antibodies. The cells were analyzed on the Beckman platform or sorted on the FACSAria III platform.

### 5.22 Flow Cytometry

After co‐culture, the lower cells were harvested via Accutase (Biolegend, 423201). Single‐cell suspensions were prepared in ice‐cold PBS and incubated with Fc receptor blocking solution (Human TruStain FcX, BioLegend, #422302;) for 10 min at room temperature to prevent nonspecific antibody binding, and stained with CD80 PE (305207) and CD206 BV421 (321125) (Biolegend) antibodies. The cells were analyzed on the Beckman platform cytoFLEX S, or sorted on the FACSAria III platform. Data acquisition was performed with appropriate fluorescence compensation controls, and analysis was conducted using CytExpert 1.0 with gating strategies based on size (FSC), granularity (SSC).

### 5.23 RNA‐seq Analysis for Co‐Cultured Macrophage Cells With SMART Technology

Following co‐culture, M2‐like macrophages were sorted and subjected to transcriptomic profiling using the SMART protocol for full‐length cDNA amplification. The resulting libraries were quantified using a Qubit 3.0 Fluorometer (Thermo Fisher, Q33216), and their size distribution was assessed on an Agilent 2100 Bioanalyzer (Agilent, G2939AA). Libraries passing quality control were sequenced on an Illumina platform with 150 bp paired‐end reads. Raw sequencing data were processed, and differential expression, along with enrichment analyses, were performed using the same bioinformatic pipeline as previously described for bulk RNA‐seq of LUAD cancer cells.

### 5.24 Co‐Culture of Recombinant Human MIF With THP‐1‐Derived Macrophages

To directly assess the immunomodulatory function of macrophage migration inhibitory factor (MIF), recombinant human MIF (rhMIF, purchased from CellSignalingTechnology, 87501S) was added to THP‐1‐derived macrophages as indicated. M1 and M2 identification were performed as described as previously.

### 5.25 Xenografted Models

To evaluate tumor progression in vivo, a syngeneic tumor model was established using six‐week‐old C57BL/6J mice, which were maintained in a specific‐pathogen‐free (SPF) facility and handled according to the institutional ethical guidelines. Lewis lung carcinoma cells (5 × 10^7^) suspended in 100 µL of phosphate‐buffered saline (PBS) with MATRIGEL (bioGenous, M315077) were subcutaneously injected into the right inguinal region of each mouse. On Day 8, when tumor volumes reached approximately 100 mm^3^, mice were randomized into two groups (5 mice per group): one receiving PBS and the other treated with the CHEK1 inhibitor. Mice in the treatment group received the CHEK1 inhibitor SRA737 (HY‐18958, MedChemExpress) daily by oral gavage at a dose of 150 mg/kg, while control mice received an equal volume of PBS. The treatment was continued for 7 days. Tumor volume was measured with a digital caliper and calculated using the formula for a prolate ellipsoid: Volume = (Length × Width^2^) / 2. Following CHEK1 inhibitor administration on Day 8, a pronounced reduction in tumor size was observed at the next measurement (Day 12). To closely capture this rapid response, measurements were subsequently taken daily from Day 13 to Day 15. At the experimental endpoint, mice were euthanized, tumors were entirely dissected, and their weights were recorded, as well as the body weight of mice. In a parallel experiment, mice bearing established LLC tumors were randomized to receive either the MIF inhibitor ISO‐1 (HY‐16692, MedChemExpress, 35 mg/kg/day, administered via intraperitoneal injection) or PBS (5/group) for a duration of 10 days. One mouse in the PBS control group died prior to the endpoint, and therefore one mouse from the treatment group was excluded to maintain matched group sizes. Tumor weight, the primary endpoint, was subsequently evaluated for a total of eight tumors (4/group).

### 5.26 Single‐Cell RNA Sequencing Analysis of Murine Tumors

Single‐cell RNA sequencing was performed to characterize the tumor immune microenvironment following CHEK1 or MIF inhibitor treatment. Tumors were dissociated into single‐cell suspensions. Libraries were constructed using the MobiCube kit on the MobiNova‐100 platform and sequenced on the DNBSEQ‐T7 (PE150). Raw FASTQ files were processed with MobiVision software (version 3.2), aligned to the GRCm39 genome, and UMI counts were quantified (processing services provided by OE Biotech Co., Ltd., Shanghai, China). Downstream analysis was conducted using Seurat for quality control, normalization, PCA, and graph‐based clustering. Cell populations were annotated by assessing the expression of canonical marker genes. Key markers included Serpinf1 and Krt8 for epithelial cells, Col1a1/Col1a2 for fibroblasts, and lineage‐specific immune markers such as Cd3 (T cells), Gzma (NK cells), and Cd68 (macrophages). Macrophage subsets were classified as M2 (Arg1^+^Cd86^−^) or M1 (Arg1^−^Cd86^+^), where a gene was considered “positive” if its normalized expression > 1, and “negative” if < 0.0001. In parallel, M1 and M2 polarization states were also evaluated based on gene set scoring using the Seurat *AddModuleScore* function. The gene modules used for scoring (M1: *Cd86*, *Cd80*, *Nos2*, *Stat1*, *Il6*, *Tnf*; M2: *Arg1*, *Thbs1*, *Fn1*) were curated from the CellMarker 2.0 database [107] and established macrophage polarization guidelines [118]. To ensure a more accurate definition, a macrophage was assigned to the M1 subset only if its M1 module score exceeded the mean score and its expression of the canonical M2 marker Arg1 was below 0.0001, consistent with experimental recommendations for distinguishing activation states. A macrophage was assigned to the M2 subset if its M2 module score exceeded the mean. T cell subsets were defined as Foxp3^+^, Ctla4^+^, or Lag3^+^Cd8^+^ based on the co‐expression of their respective marker genes (expression > 1) within the annotated T cell population.

### 5.27 Analysis of Clinical Single‐Cell RNA Sequencing Cohort

To investigate the association between CHEK1 expression in tumor cells and clinical outcomes to immunotherapy, we analyzed a published single‐cell RNA‐seq dataset (GSE12345 [88], Table S9) from 33 lung adenocarcinoma patients treated with anti‐PD‐1 therapy. The proportion of CHEK1‐positive cancer cells was calculated for each patient, and its correlation with treatment response (responder vs non‐responder) was evaluated using a generalized linear model, adjusting for age, gender, and smoking history. The model's discriminatory power was assessed by the area under the receiver operating characteristic (ROC) curve.

### 5.28 Statistical Analysis

All statistical analyses were performed using R (version 4.0.5). Statistical significance was defined as *p* < 0.05. Details of the statistical tests and exact *p*‐values for each experiment are provided in the figure legends and the corresponding Methods section. Additional visualizations (e.g., violin plots, box plots, UMAPs, and heatmaps) were generated using the R packages ggplot2 (version 3.1.1), ggpubr (version 0.6.1), pheatmap (version 1.0.12), and Seurat (version 4.4.0).

## Author Contributions

FBM, TW, TL, PPZ, BJS, and HSB conceived and designed the study. TW collected the data and performed the analysis. PPZ, XLZ, KLL, TL, TW, and FBM designed the experiments. YQD, TL, HYZ, RYZ, and XTY performed the experiments. ZPZ collected and arranged the scRNA‐seq data. XJS, HSB, DW, YL, and LBW collected the clinical samples. FBM, TL, PPZ, and TW wrote and edited the paper. All authors improved the manuscript and approved the submission.

## Conflicts of Interest

The authors declare no conflicts of interest.

## Supporting information




**Supporting File 1**: advs74522‐sup‐0001‐SuppMat.docx.

**Supporting File 2**: advs74522‐sup‐0002‐Supplementary Tables S1‐S9.zip.

## Data Availability

The data that support the findings of this study are available from the corresponding author upon reasonable request.
