# Supplementary material for: Pancancer Fine‐Mapping of Mutational Intolerance Identifies CHEK1 as an Immunosuppressive Driver in Lung Adenocarcinoma
Source: Adv Sci (Weinh). 2026 Feb 21;13(25):e21265. doi: 10.1002/advs.202521265 (PMC13137788; doi:10.1002/advs.202521265)
Supplement: Supplementary file 1 — Supporting File 1: advs74522‐sup‐0001‐SuppMat.docx. [file ADVS-13-e21265-s002.docx]

**Online Supplementary Content**

[**Figure S1.** Significantly mutated genes in each type of healthy and tumor tissues. 1](#_Toc221620952)

[**Figure S2.** Cell cycle-related MIGs exhibited significant prognostic implications across various cancer types 2](#_Toc221620953)

[**Figure S3.** Cell cycle-related MIGs may have an impact on the tumor microenvironment in multiple cancer types. 3](#_Toc221620954)

[**Figure S4.** Pan-cancer cell type annotation and identification of immune-suppressive cell populations 4](#_Toc221620955)

[**Figure S5.** Additional evidence supporting the tumor-promoting function for survival-related MIGs in LUAD. 5](#_Toc221620956)

[**Figure S6.** Summary of RNA-seq analysis comparing CHEK1/DHX37/CDC45 knockdown LUAD cancer cells to control cells. 6](#_Toc221620957)

[**Figure S7.** MIGs influence LUAD cell stemness via regulating cell cycle and telomere function 7](#_Toc221620958)

[**Figure S8.** Single-cell transcriptomic profiling of MIG perturbation using a CRISPR base-editing library 8](#_Toc221620959)

[**Figure S9.** Cell type annotation of immune cell subpopulations and identification of immune suppressive cell subpopulations in LUAD. 9](#_Toc221620960)

[**Figure S10.** Evidence for the CHEK1-p53-MIF axis. 11](#_Toc221620961)

[**Figure S11.** CHEK1 in tumor cells shapes an immunosuppressive microenvironment 12](#_Toc221620962)

[**Figure S12.** Targeting the CHEK1-MIF axis reverses immunosuppression and is linked to poor immunotherapy response 13](#_Toc221620963)

[**Figure S13.** Spatial and functional analyses of CHEK1/MIF in tumor-macrophage interactions 15](#_Toc221620964)

## **Figure S1.** Significantly mutated genes in each type of healthy and tumor tissues.

**
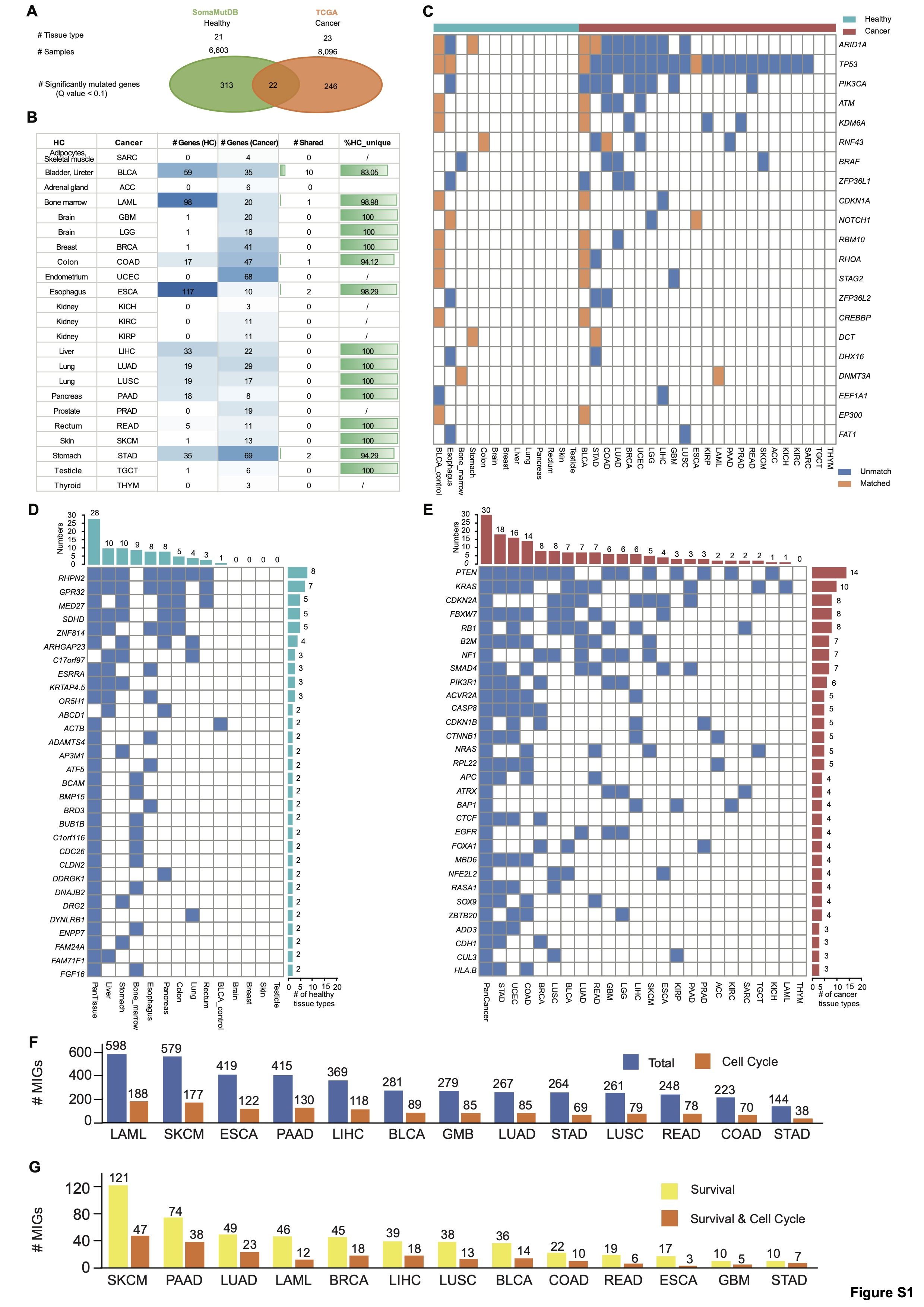
**

(**A**) Summary of SMGs identified in healthy and tumor tissues. (**B**) Summary of shared SMGs between each type of tumor and corresponding healthy tissues. Distribution of shared (**C**) and specific (**D, E**) SMGs across various healthy and cancer tissues. (**F**) Enrichment of MIGs in tumor drug targets and the distribution of drug-related MIGs across various cancer types. (**G**) Distribution of survival-related MIGs (Survival P < 0.05) and cell cycle-related survival MIGs across different cancer types. MIG, mutation-intolerant gene.

## **Figure S2.** Cell cycle-related MIGs exhibited significant prognostic implications across various cancer types


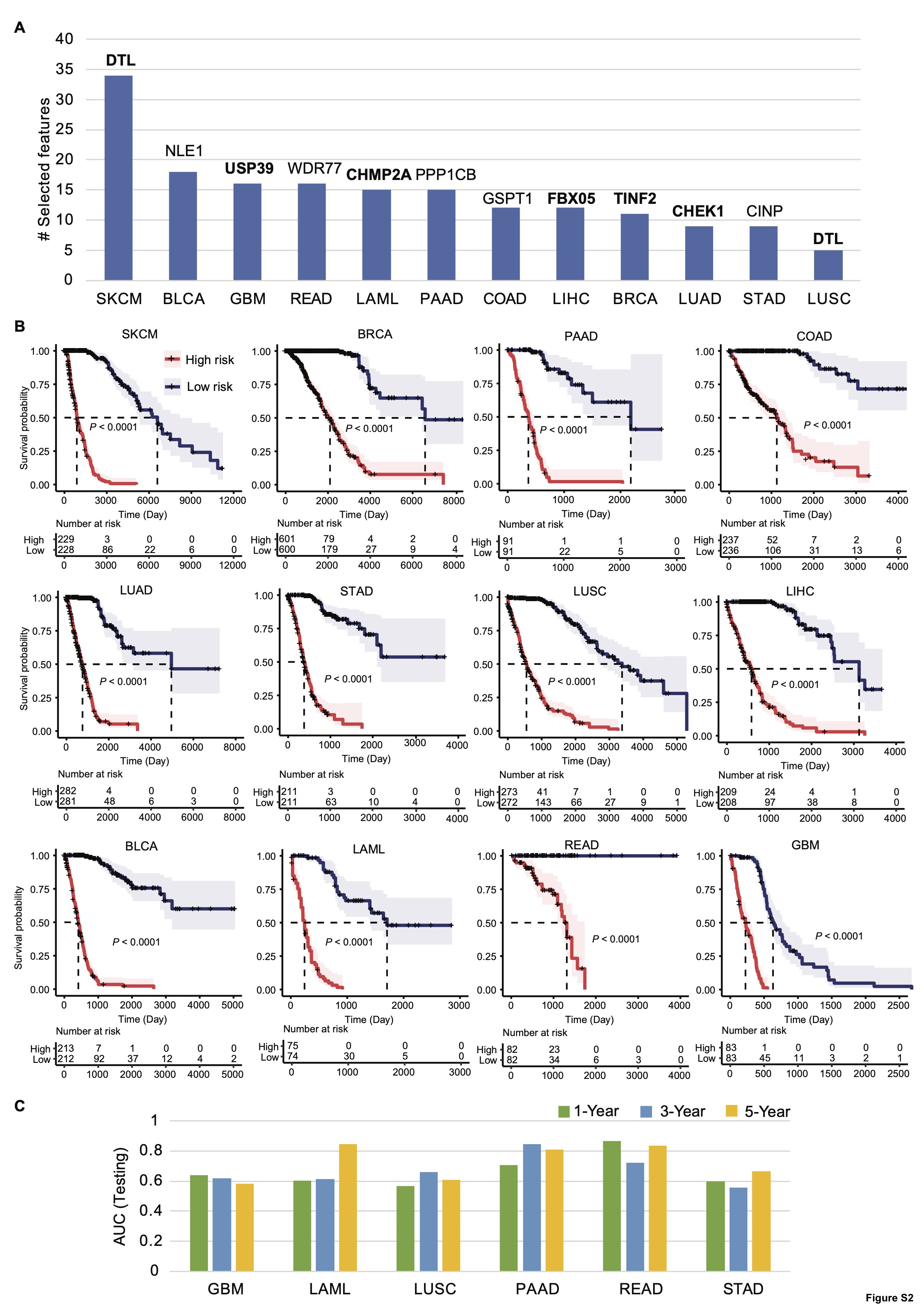


(**A**) Summary of MIGs identified by the constructed prognostic model in each cancer type. (**B**) Patients with a lower CMPS exhibited significantly longer survival compared to those with a higher CMPS. CMPS, CellCycle MIG-related prognostic risk score. The median value of CMPS was used for grouping patients into CMPS high or low-risk groups in each cancer type. The Log-rank test was used for P value calculation. (**C**) The prognostic potential of MIGs in remaining testing cohorts across six cancer types (This figure supplements Figure 2C by presenting the AUC values for the six cancer cohorts that were not included in the main figure).

## **Figure S3.** Cell cycle-related MIGs may have an impact on the tumor microenvironment in multiple cancer types.


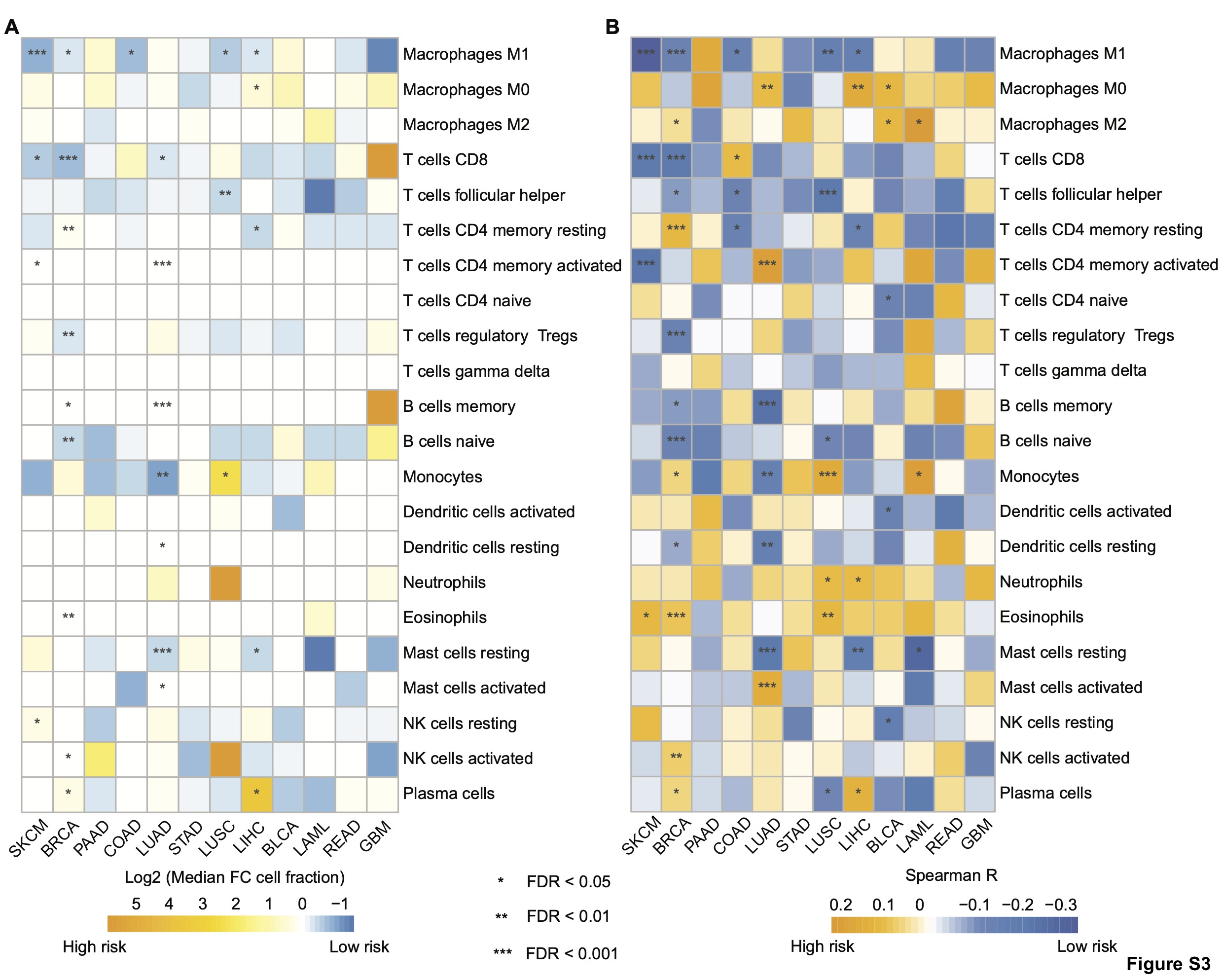


(**A**) Results of the differential abundance analysis of each type of immune cells between the high and low groups of CMPS. The Wilcoxon-rank sum test was used for P value calculation. (**B**) Correlation results between the abundance of each type of immune cell and CMPS scores across various cancer types. The spearman correlation test was used for P value calculation. CMPS, CellCycle MIG-related prognostic risk score. The "p.adjust" function in R using the "BH" method was utilized to compute the adjusted p-values for multiple comparisons. *, FDR < 0.05; **, FDR < 0.01; ***, FDR < 0.001.

## **Figure S4.** Pan-cancer cell type annotation and identification of immune-suppressive cell populations


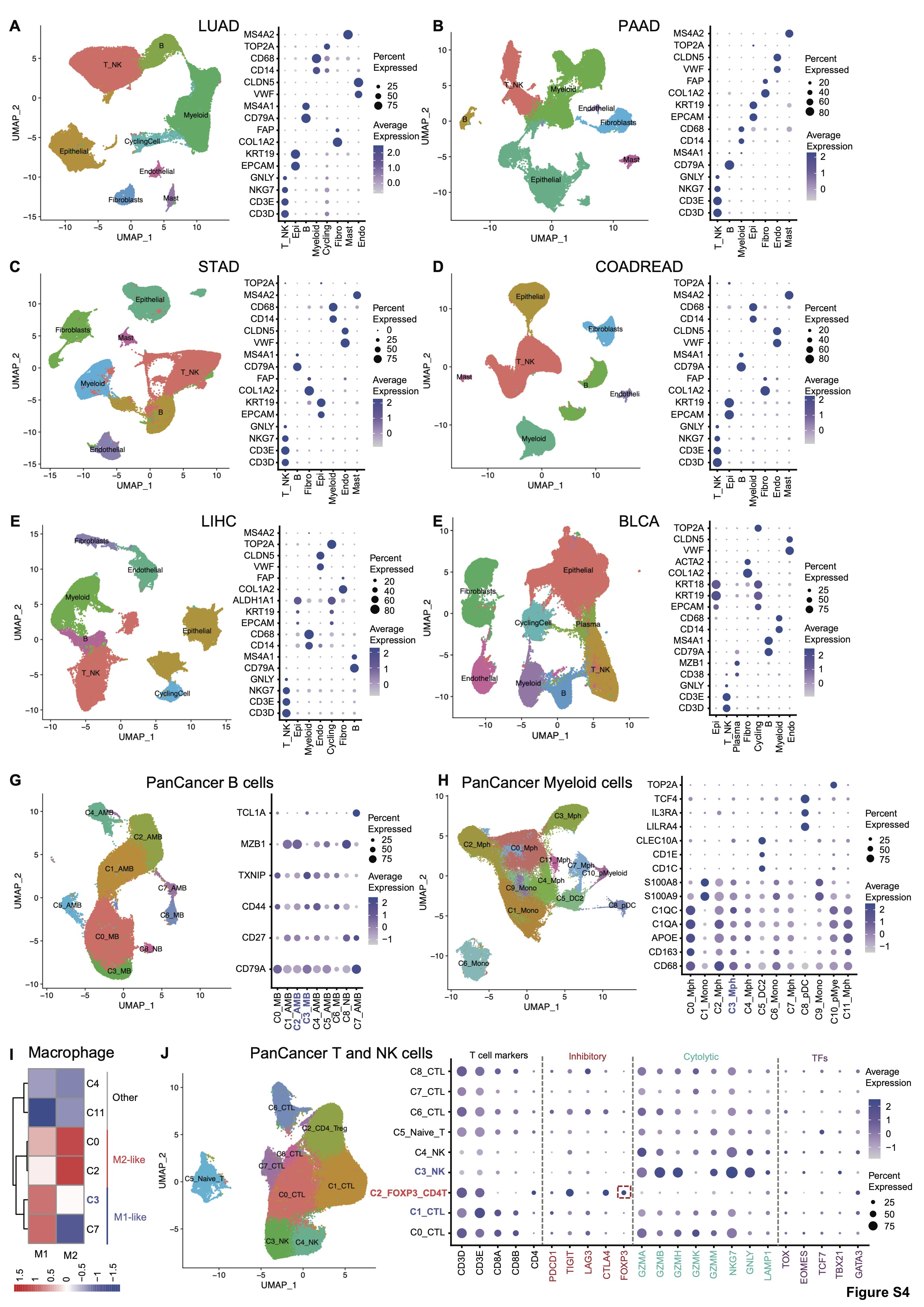


(**A-F**) The major cell population UMAP plots and their marker gene expression dot plots for LUAD, PAAD, STAD, COADREAD, LIHC, and BLCA. (**G-H**) The subpopulation UMAP plots and their marker gene expression dot plots for B cells, and myeloid cells. (**I**) Estimating the functional features of the macrophage cell cluster. The M1 and M2 macrophage scores were calculated based on the mean expression of M1 and M2 marker genes listed in Supplementary Table 13. **(J)** The subpopulation UMAP plots and their marker gene expression dot plots for T and NK cells. Epi, epithelial cells; Endo, endothelial cells; Fibro, fibroblasts; MB, Memory B cells; AMB, Activated memory B cells; NB, Naive B cells; Mph, macrophage; Mono, monocytes; pMye, proliferating myeloid cell; pDC, plasmacytoid dendritic cells; DC2, type 2 dendritic cells; CTL, cytotoxic CD8 T cells; Treg, regulatory T cells; NK, Natural Killer; The size of the dot represents the percentage of expressed cells; The color of the dot represents the scaled average expression level of corresponding marker gene;

## **Figure S5.** Additional evidence supporting the tumor-promoting function for survival-related MIGs in LUAD.


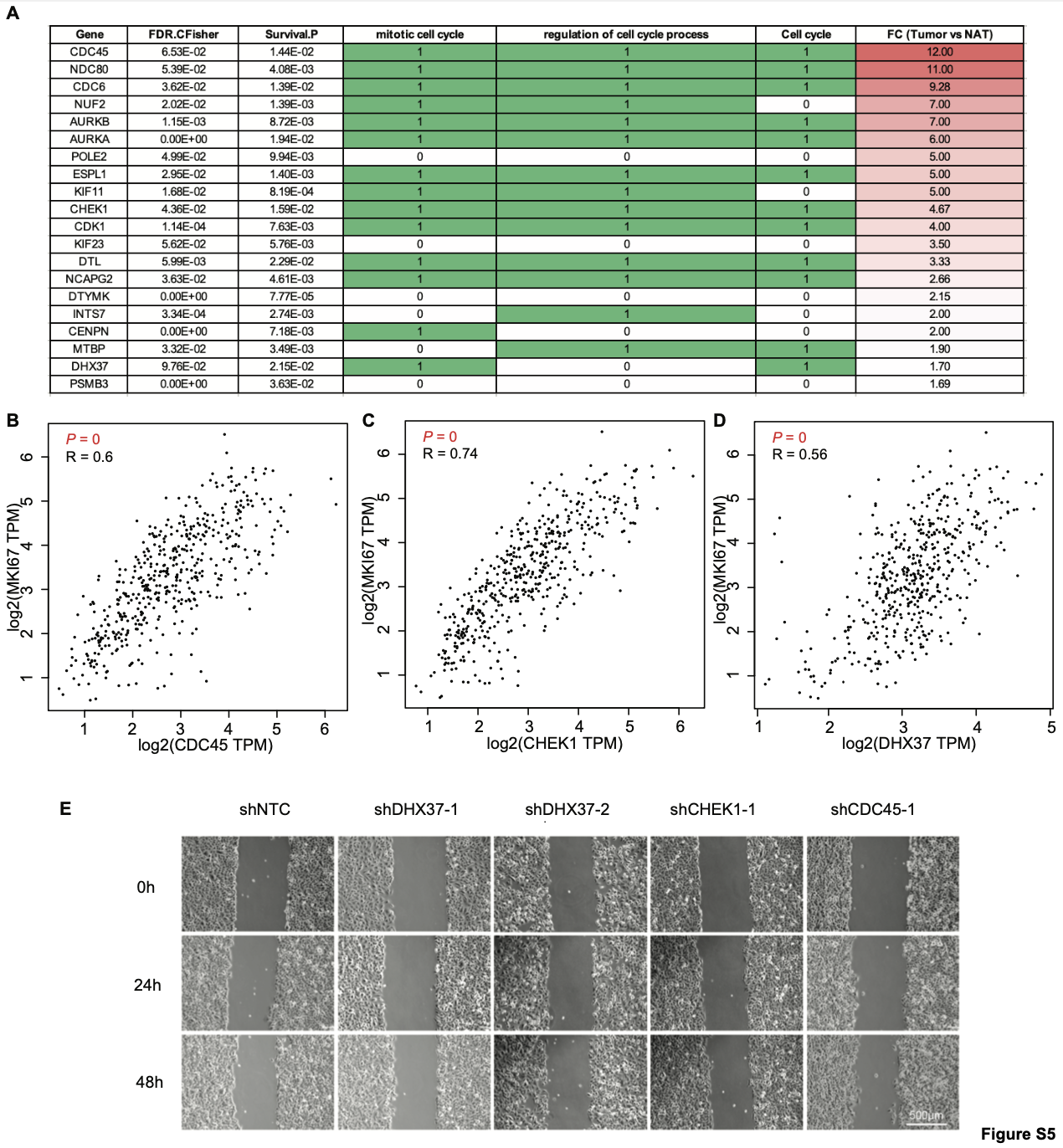


(**A**) Top 20 survival-related MIGs in LUAD, ranked by the fold change of their expression between tumor and NAT. (**B-D**) Correlation plots generated by the GEPIA2 database showing the relationship between CDC45, CHEK1, and DHX37 with the tumor progression marker gene KI-67. (**E**) The wound healing assay demonstrated that the downregulation of both DHX37 and CHEK1 can reduce the migration ability in A549 cancer cells. Scale bar = 500 μm. TPM, transcripts per million mapped reads.

## **Figure S6.** Summary of RNA-seq analysis comparing CHEK1/DHX37/CDC45 knockdown LUAD cancer cells to control cells.


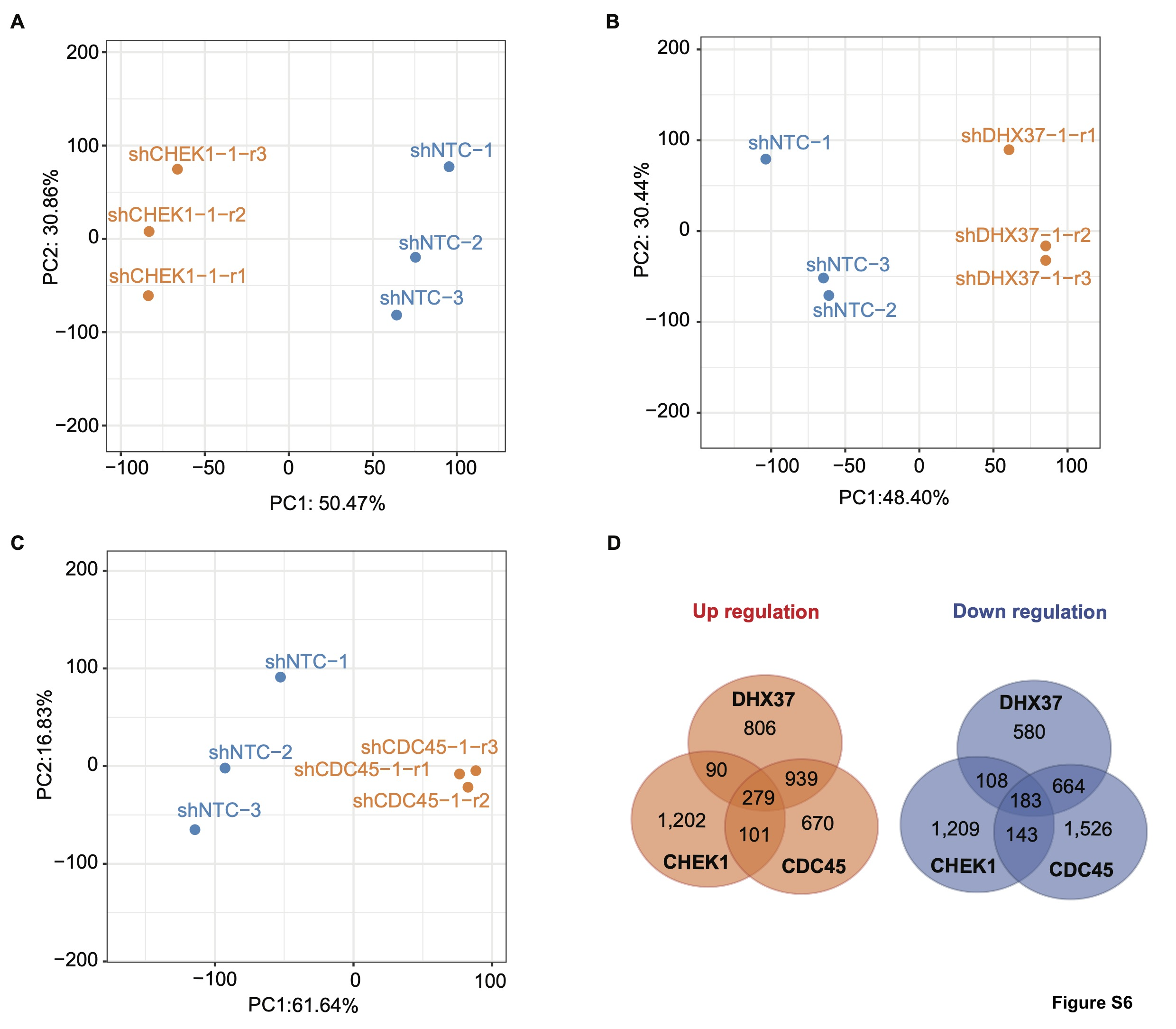


(**A-C**) Principal component analysis (PCA) revealed well-clustered samples in both the control group (shNTC) and the CHEK1/DHX37/CDC45 gene knockdown (shCHEK1/shDHX37/shCDC45) group. shNTC, non-targeting control shRNA. (**D**) Comparison of significantly differentially expressed genes among three above comparisons. Fold change > 1.5 and FDR < 0.05 are considered as of significantly differential expression.

## **Figure S7.** MIGs influence LUAD cell stemness via regulating cell cycle and telomere function

**
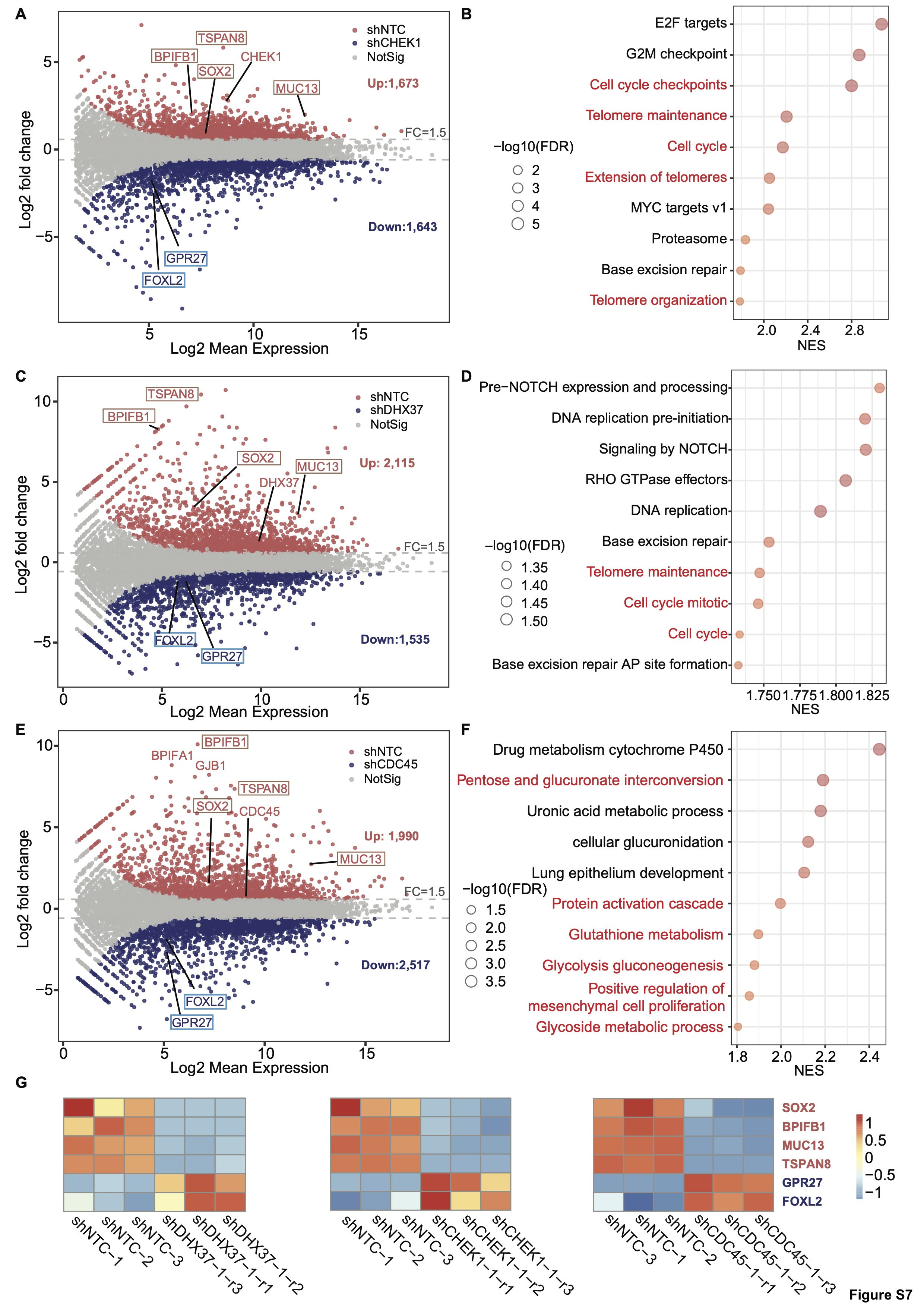
**

(**A**) Volcano plot for differentially expressed genes (DEGs) between the shNTC and shCHEK1 groups. (**B**) GSEA enrichment analysis of protein-coding genes ranked by fold change between the shNTC and shCHEK1 groups. (**C**) Volcano plot for differentially expressed protein-coding genes between the shNTC and shDHX37 groups. (**D**) GSEA enrichment analysis of protein-coding genes ranked by fold change between the shNTC and shDHX37 groups. (**E**) Volcano plot depicting differentially expressed protein-coding genes between the shNTC and shCDC45 groups. (**F**) GSEA enrichment analysis of protein-coding genes ranked by fold change between the shNTC and shCDC45 groups. (**G**) A heatmap illustrating significantly differentially expressed protein-coding genes shared among the above three comparisons. Significantly DEGs were defined with fold change > 1.5 and FDR < 0.05. vs., versus; shNTC, non-targeting control shRNA.

## **Figure S8.** Single-cell transcriptomic profiling of MIG perturbation using a CRISPR base-editing library


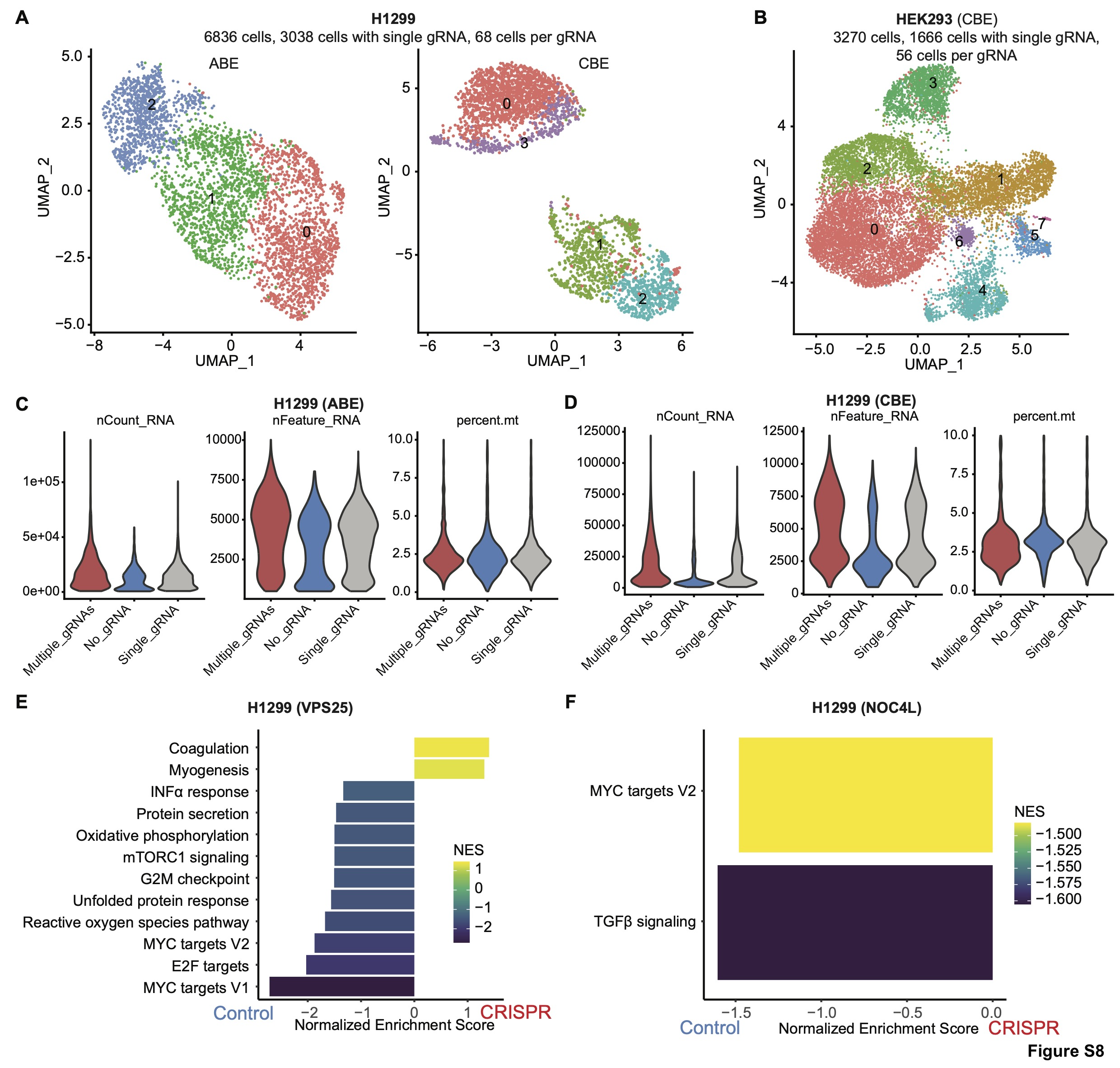


**(A-B)**. Single-cell transcriptome visualization (UMAP) of H1299 **(A)** and HEK293 **(B)** cells transduced with a MIG-targeting CRISPR base-editing library. **(B)** The UMAP plots for MIG perturbation in HEk293 cells. **(C-D)** Quality control metrics for the H1299 ABE and CBE libraries, Violin plots showing nCount_RNA, nFeature_RNA, and percent.mt for the ABE library **(C)** and the CBE library **(D)**. **(E)** or NOC4L **(F)** in H1299 cells downregulates oncogenic pathways, as shown by GSEA. **(E-F)** Targeting VPS25 **(E)** or NOC4L **(F)** in H1299 cells downregulates oncogenic pathways, as shown by GSEA.

## **Figure S9.** Cell type annotation of immune cell subpopulations and identification of immune suppressive cell subpopulations in LUAD.


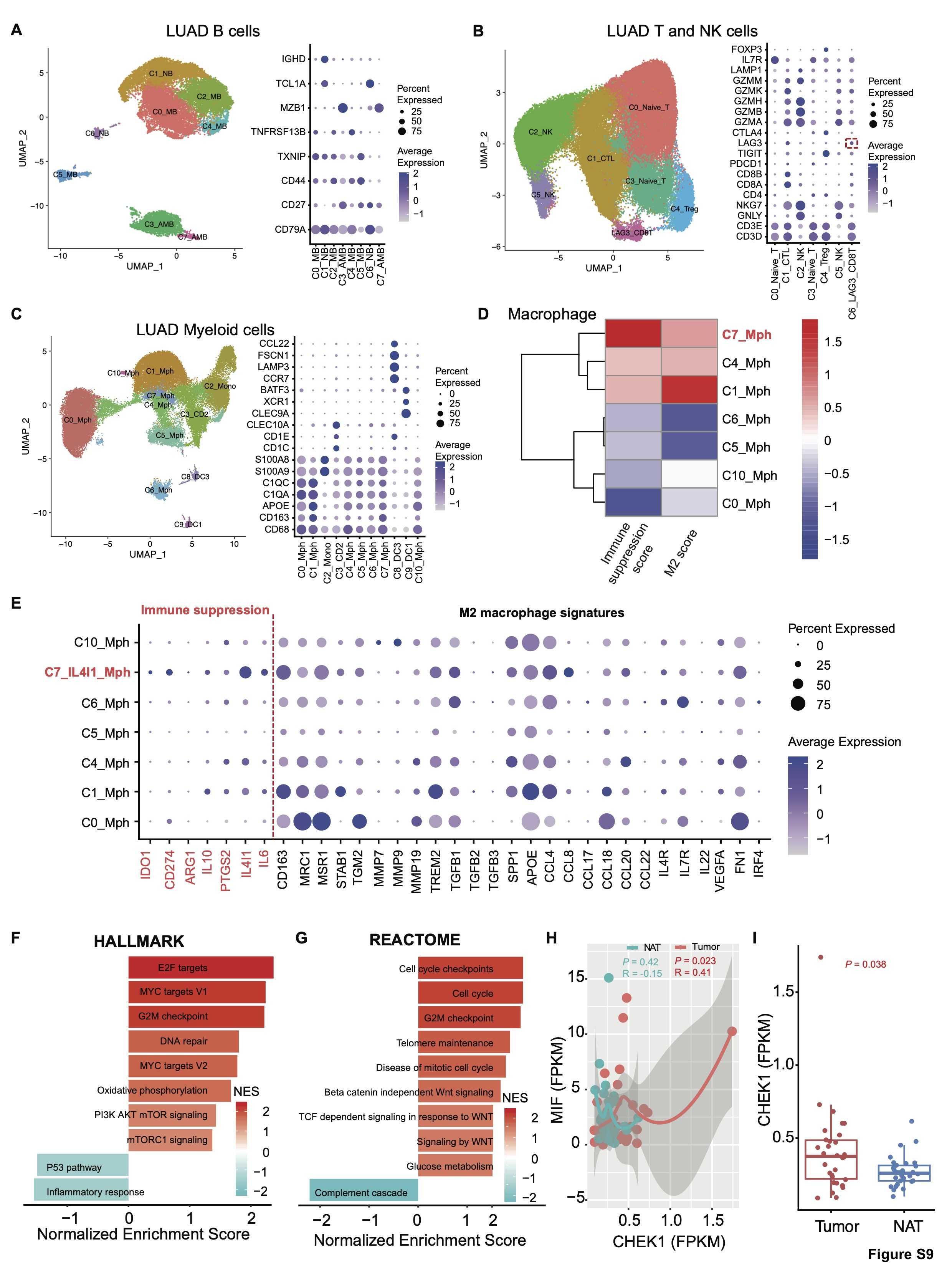


**(A-C)** The subpopulation UMAP plots and corresponding marker gene expression dot plots for LUAD B cells, LUAD T and NK cells, and LUAD myeloid cells. **(D)** Heatmap of the immune suppression score and M2 macrophage score for the macrophage cell cluster. The immune suppression and M2 macrophage scores were calculated based on the mean expression of corresponding marker genes in (D). **(E)** Dot plot of the immune suppression and M2 macrophage marker genes for the macrophage cell cluster. MB, Memory B cells; AMB, Activated memory B cells; NB, Naive B cells; CTL, cytotoxic CD8 T cells; Treg, regulatory T cells; NK, Natural Killer; Mph, macrophage; Mono, monocytes; DC1, type 1 dendritic cells; DC2, type 2 dendritic cells; DC3, type 3 dendritic cells; The size of the dot represents the percentage of expressed macrophage cells for each marker gene, while the color of the dot indicates the scaled average expression level of each marker gene.(**F-G**) GSEA enrichment analysis of the significantly dysregulated HALLMARKs and REACTOME pathways in CHEK1^+^ LUAD cancer cluster. The color represented the normalized enrichment score (NES). (**H**) Scatter plots demonstrate differential CHEK1-MIF co-expression patterns in LUAD tumors versus matched normal tissues (n=28, paired samples from an independent cohort (our unpublished data)). Tumors (red) show significant positive correlation (Pearson's R=0.41, linear model P=0.023), while normal adjacent tissue (green) displays no significant association (R=-0.15, P=0.42). Solid lines indicate linear regression fits with 95% confidence bands (shaded). (**I**) Boxplot shows significant higher expression of CHEK1 in LUAD tumors (red; n=28) than normal adjacent tissues (blue; n=28; P=0.038, Wilcoxon signed-rank test).

## **Figure S10.** Evidence for the CHEK1-p53-MIF axis.


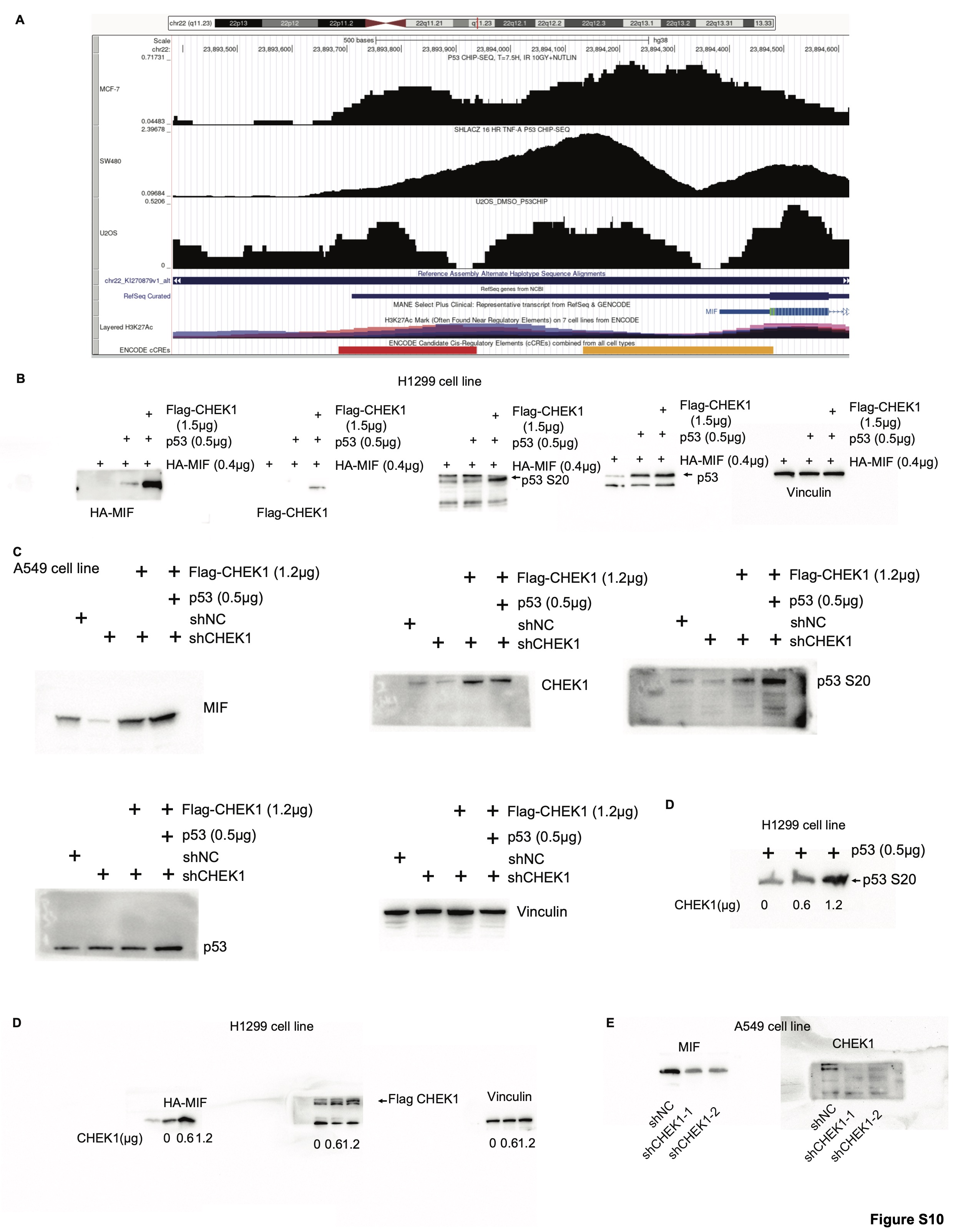


**(A)** H3K27Ac-marked MIF regulatory elements are bound by TP53 across cancer cell lines (Source: Cistrome DB (2019). Screenshot of integrated ChIP-seq tracks shows co-occupancy of TP53 and H3K27ac at canonical MIF regulatory loci.). **(B)** Full, uncropped Western blot images corresponding to Figure 6H. **(C)** Full, uncropped Western blot images corresponding to Figure 6J. **(D)** Full, uncropped Western blot images corresponding to Figure 6G. (**E**) Full, uncropped Western blot images corresponding to Figure 7G.

## **Figure S11.** CHEK1 in tumor cells shapes an immunosuppressive microenvironment

**
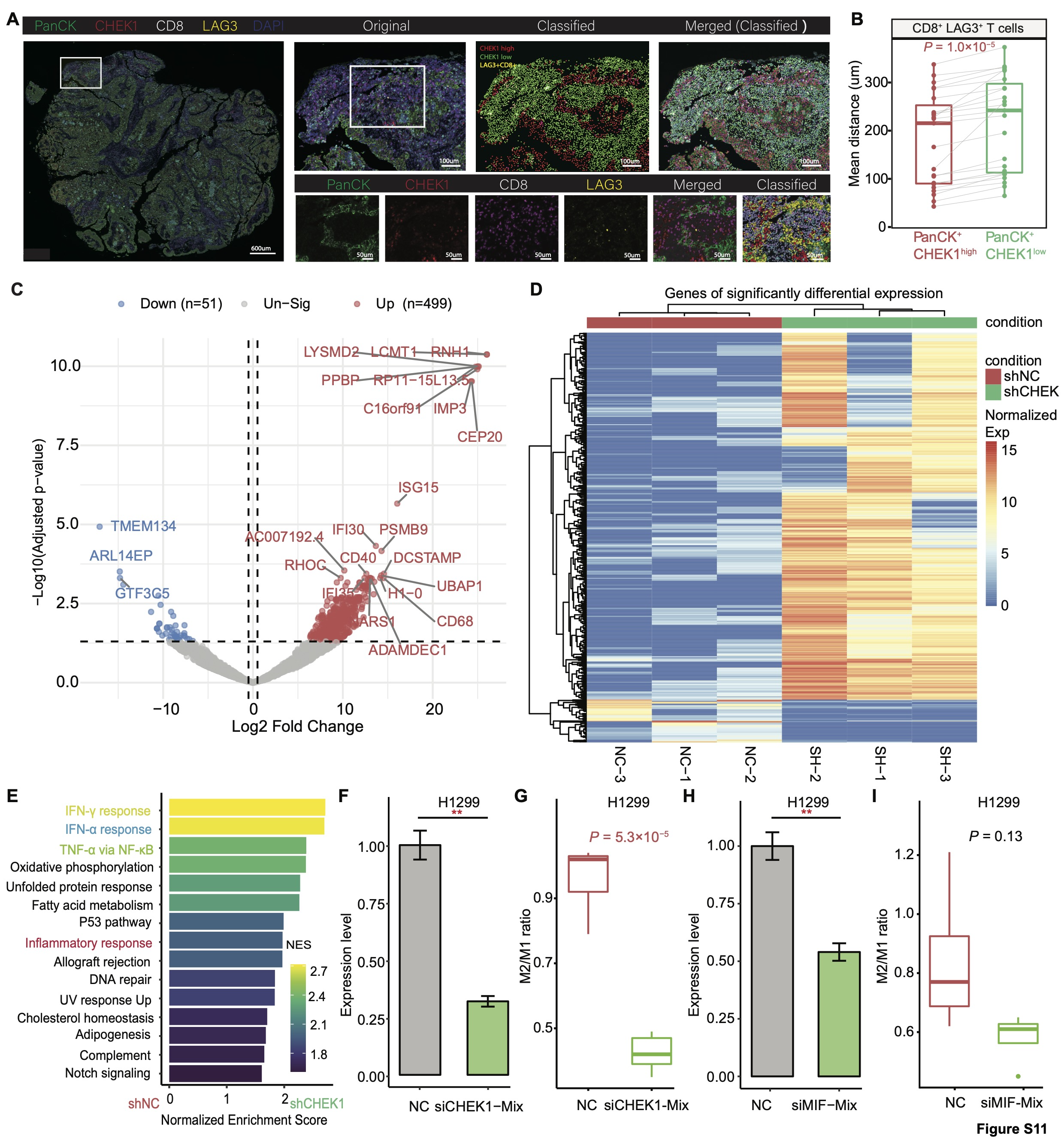
**

**(A)** Representative images were shown for mIF of PanCK/CHEK1 (CHEK1high/low cancer cells), and CD8/LAG3 (LAG3+CD8+ T cells) (n = 6). **(B)** Comparison of the average spatial distances between CHEK1high cancer cells or CHEK1low cancer cells to LAG3+CD8+ T cells (number of ROI=23). **(C)** Volcano plot and **(D)** Heatmap of differentially expressed genes in macrophages co-cultured with shCHEK1 versus shNC tumor cells (n=3; |log₂FC|> 1, adj. p < 0.05). **(E)** GSEA results of macrophages co-cultured with shCHEK1 versus shNC tumor cells. **(F)** Quantification of CHEK1 expression by qPCR in H1299 cells transfected with siCHEK1 versus siNC controls. **(G)** Flow cytometry analysis of macrophage markers (CD206, CD80) following co-culture with siNC or siCHEK1 H1299 LUAD cells. **(H)** Quantification of MIF expression by qPCR in H1299 cells transfected with siMIF versus siNC controls. **(I)** Flow cytometry analysis of macrophage markers (CD206, CD80) following co-culture with siNC or siMIF H1299 LUAD cells. Statistical analysis was calculated using the two-tailed t-test.

## **Figure S12.** Targeting the CHEK1-MIF axis reverses immunosuppression and is linked to poor immunotherapy response


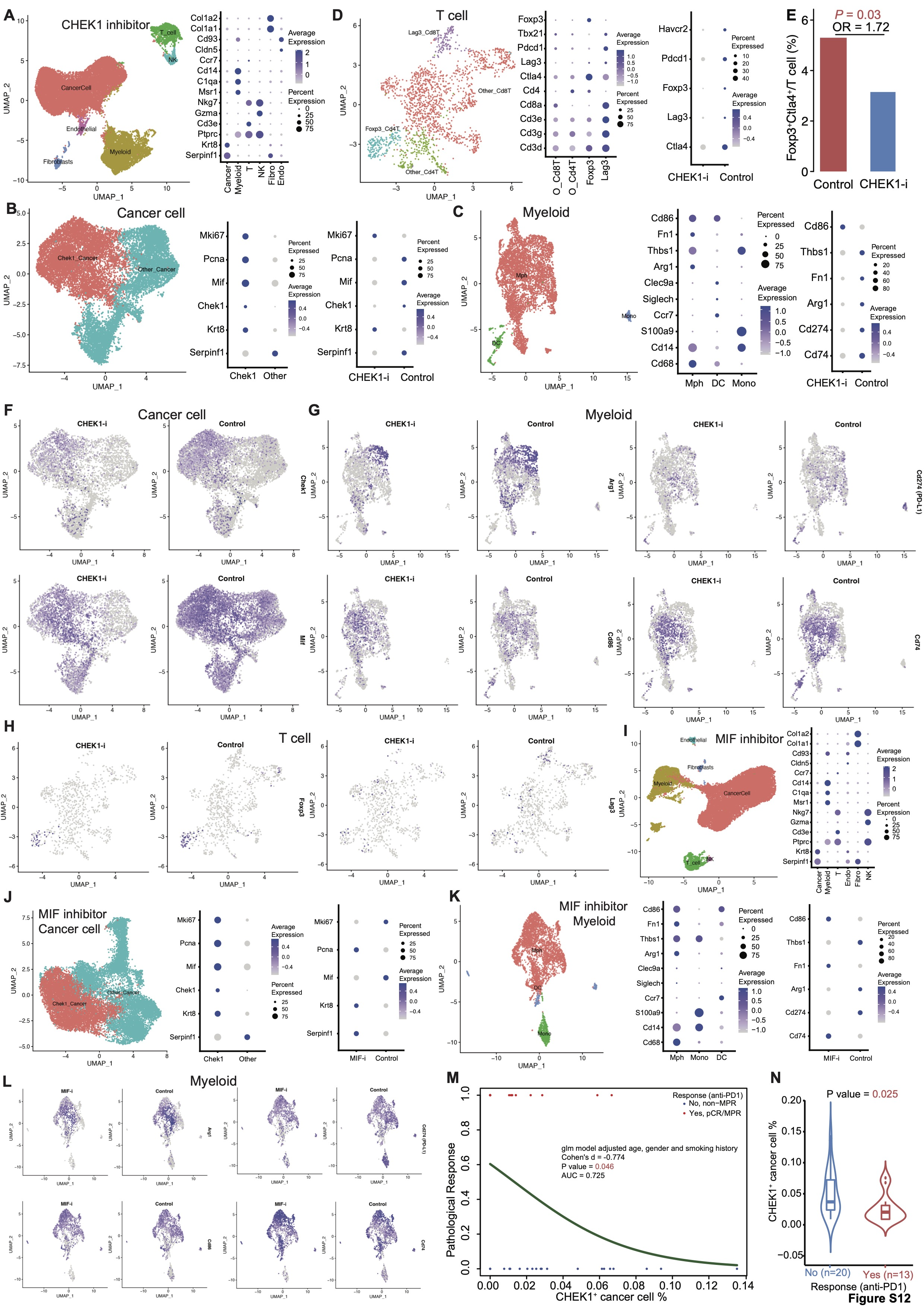


(**A-H**) Single-cell profiling of CHEK1-inhibitor treated tumors. (A) UMAP visualization of major cell populations from integrated scRNA-seq data (combined CHEK1 inhibitor treatment and control groups). (B-D) Subtype analysis of cancer, myeloid, and T cells. For each major celltype: (left) UMAP visualization of cell subtypes; (middle) dot plot showing expression of canonical subtype-defining marker genes; (right) dot plot comparing expression of key genes of interest between the CHEK1 inhibitor-treated and control groups. (E) Percentages of Foxp3⁺Ctla4⁺ T cells in the CHEK1 inhibitor group versus their respective PBS controls. (F-H) Expression patterns of key genes in cancer, myeloid, and T cells. Feature plots showing treatment-control comparisons for genes of interest. (**I-L**) Single-cell profiling of MIF-inhibitor treated tumors. (I) UMAP visualization of major cell populations from integrated scRNA-seq data (combined MIF inhibitor treatment and control groups). (J-K) Subtype analysis of cancer and myeloid cells. (L) Feature plots showing treatment-control comparisons for key genes in myeloid cells. **(M)** A generalized linear model adjusted for age, gender, and smoking history was used to evaluate the association between the CHEK1‑positive cancer cell ratio and treatment response (n=33). **(N)** CHEK1-positive cancer cell ratio in LUAD patients responding versus not responding to anti-PD-1 therapy (two-sided t-test).

## **Figure S13.** Spatial and functional analyses of CHEK1/MIF in tumor-macrophage interactions


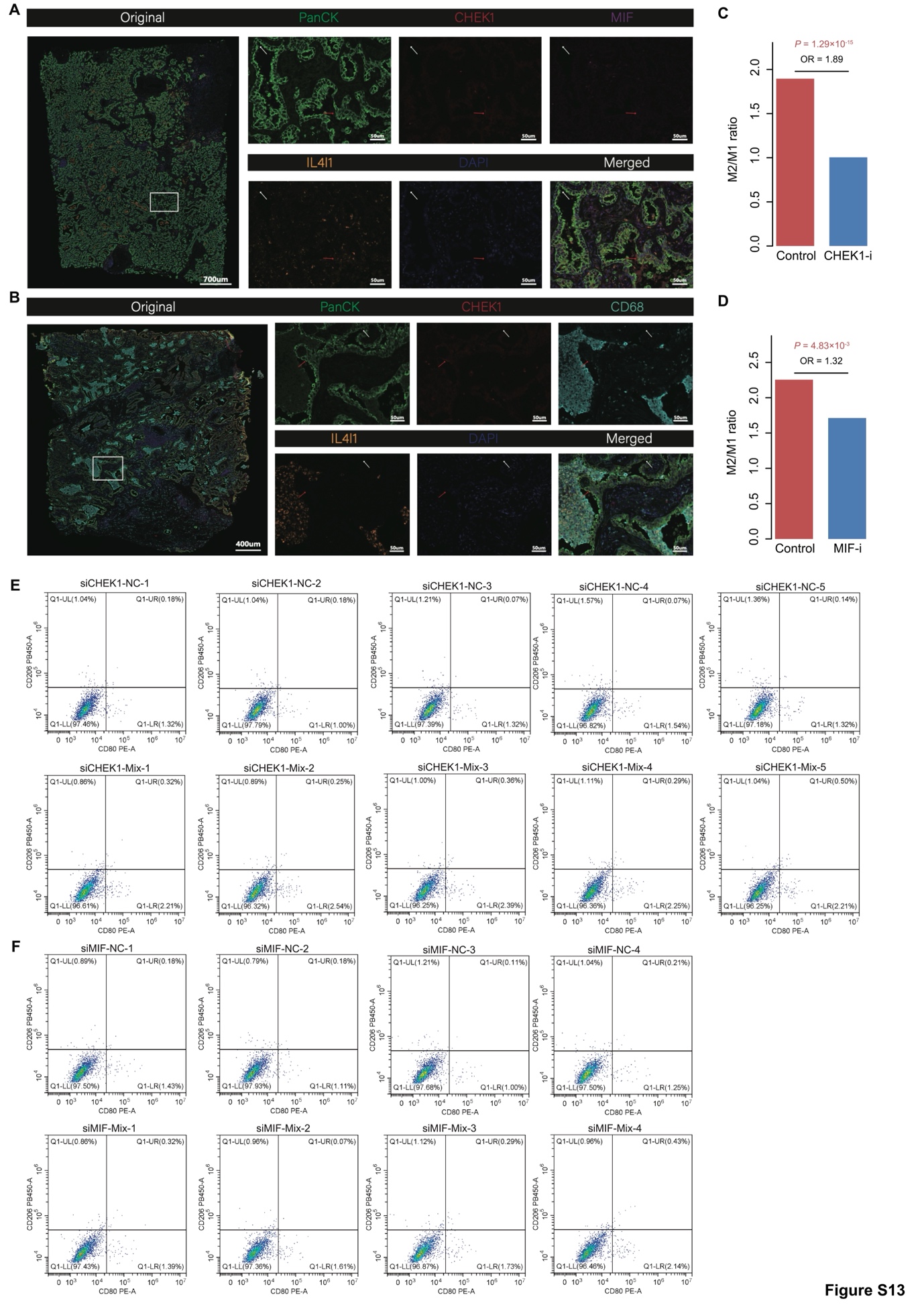


**(A)** Representative immunofluorescence images showing the co-localization of CHEK1 and MIF in cancer cells; **(B)** Representative immunofluorescence images depicting the spatial distribution of CHEK1 expressing cancer cells relative to M2-like macrophages. White arrows indicate regions with low CHEK1 expression; red arrows denote regions with high CHEK1 expression. **(C, D)** The M2/M1 macrophage ratio in tumors treated with the CHEK1 inhibitor **(C)** or the MIF inhibitor **(D)**, compared to PBS‑treated controls. M1 and M2 subsets were defined based on gene‑set scoring. **(E, F)** Fow cytometry plots showing the sorting of M1 and M2 macrophages from THP-1 cells co-cultured with H1299 cells transfected with siCHEK1 **(E)** or siMIF **(F)**, compared to control transfections. These data correspond to and support the quantitative findings presented in Figure S11, panels G and I.
